# Soluble individual metal atoms and ultrasmall clusters catalyze key synthetic steps of a natural product synthesis

**DOI:** 10.1038/s42004-024-01160-z

**Published:** 2024-04-04

**Authors:** Silvia Rodríguez-Nuévalos, Miguel Espinosa, Antonio Leyva-Pérez

**Affiliations:** Instituto de Tecnología Química (UPV-CSIC), Universidad Politècnica de València-Consejo Superior de Investigaciones Científicas, Avda. de los Naranjos s/n, 46022 Valencia, Spain

**Keywords:** Homogeneous catalysis, Natural product synthesis, Synthetic chemistry methodology

## Abstract

Metal individual atoms and few-atom clusters show extraordinary catalytic properties for a variety of organic reactions, however, their implementation in total synthesis of complex organic molecules is still to be determined. Here we show a 11-step linear synthesis of the natural product (±)-Licarin B, where individual Pd atoms (Pd_1_) catalyze the direct aerobic oxidation of an alcohol to the carboxylic acid (steps 1 and 6), Cu_2-7_ clusters catalyze carbon-oxygen cross couplings (steps 3 and 8), Pd_3-4_ clusters catalyze a Sonogashira coupling (step 4) and Pt_3-5_ clusters catalyze a Markovnikov hydrosylilation of alkynes (step 5), as key reactions during the synthetic route. In addition, the new synthesis of Licarin B showcases an unexpected selective alkene hydrogenation with metal-free NaBH_4_ and an acid-catalyzed intermolecular carbonyl-olefin metathesis as the last step, to forge a *trans*-alkene group. These results, together, open new avenues in the use of metal individual atoms and clusters in organic synthesis, and confirm their exceptional catalytic activity in late stages during complex synthetic programmes.

## Abstract (web summary)

Individual metal atoms and few-atom metal clusters have shown promising catalytic activities, however, their exploitation in the total synthesis of complex organic molecules remains underexplored. Here, the authors develop a total synthesis of the bioactive natural product (±)-Licarin B involving key steps catalyzed by soluble individual Pd atoms and Cu/Pd/Pt clusters, achieving an 11-step linear synthesis and overall yield of 13.1%.

## Introduction

The eruption during the last ten years of single atoms catalysts (SACs)^1^ and catalytic few-atom metal clusters^2^ with exceptional catalytic activity (thousand to millions of turnover numbers) for different reactions have brought the organic synthetic community to adopt them for a variety of transformations^3–8^, including biologically active compounds^3,4,9^, since the metal efficiency of these ultrasmall catalytic species can in many cases be higher than classical organometallic complexes^9^. Significant progresses have been achieved in single and few-atom metal-catalyzed hydrogenation, oxidation, hydroaddition and cross-coupling reactions, to name a few, covering also from mild click chemistry to harsh radical C–H activation reactions^10^. Therefore, the time has come to test these single and few metal atom catalysts in long synthetic routes, from early to late stages, in order to confirm that the extraordinary catalytic activity of these ligand-free subnanometric metal species in benchmark reactions is maintained for structurally elaborated organic substrates. It is noteworthy to remark that the SACs and few-atom metal clusters employed here as catalysts are present in solution, not supported, thus we will name the SACs as “individual metal atoms”.

**Fig. 1:**
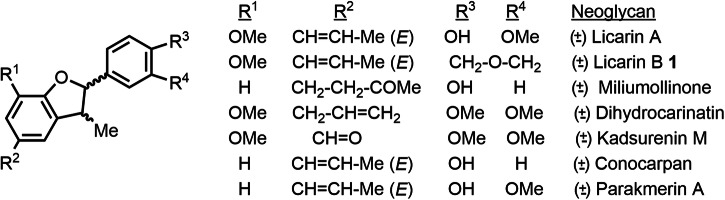
Neolignan natural products. General structure and representative examples of biologically-active dehydrobenzofurane-containing neolignan natural products. Some of these structures have been barely accomplished by total synthesis.

Lignans and neolignans constitute a huge family of natural products^11^ with a plethora of well-known pharmaceutical properties, ranging from neuroprotective^12,13^ to antiviral^14^ activity. More and more structures are found each year, and only in the last decade >500 members of the (neo)lignan family have been isolated, structurally characterized and biologically determined^11,15,16^. Within the neolignan family, the dehydrobenzofurane-containing sub-family can be considered one of the most numerous and relevant, and Fig. 1 shows representative natural products of this kind. It can be seen that the dehydrobenzofurane core is decorated with different functional groups, to achieve an array of natural products with diverse biological functions. However, in contrast to the great interest arisen by these compounds in pharma, the number of synthesis for most of them is very limited or just non-existent.

Licarin B ( ± )-**1** is a prominent member of the dehydrobenzofurane neolignan family with antibacterial^17^, antidiabetic^18,19^, antiviral^20^ and neuroprotective effects^21^. Its structure in racemic form (Fig. 1) was confirmed by total synthesis more than three decades ago (the last synthesis in 1991)^21,22^ employing the synthetic toolkit available at that time, i.e., non-catalytic procedures and extensive use of protecting groups, with accumulated yields as low as 2.5% after ~10 linear-steps^23^. As far as we know, any other synthesis of (±)-**1** has not been reported since then^11^, and this lack of modern synthetic methodologies for (±)-**1** is indeed extensible to many benzofurane (neo)lignans^24,25^. Therefore, the design of a general synthesis of benzofurane (neo)lignans based on modern catalytic reactions is still pending for the synthetic organic community.

**Fig. 2:**
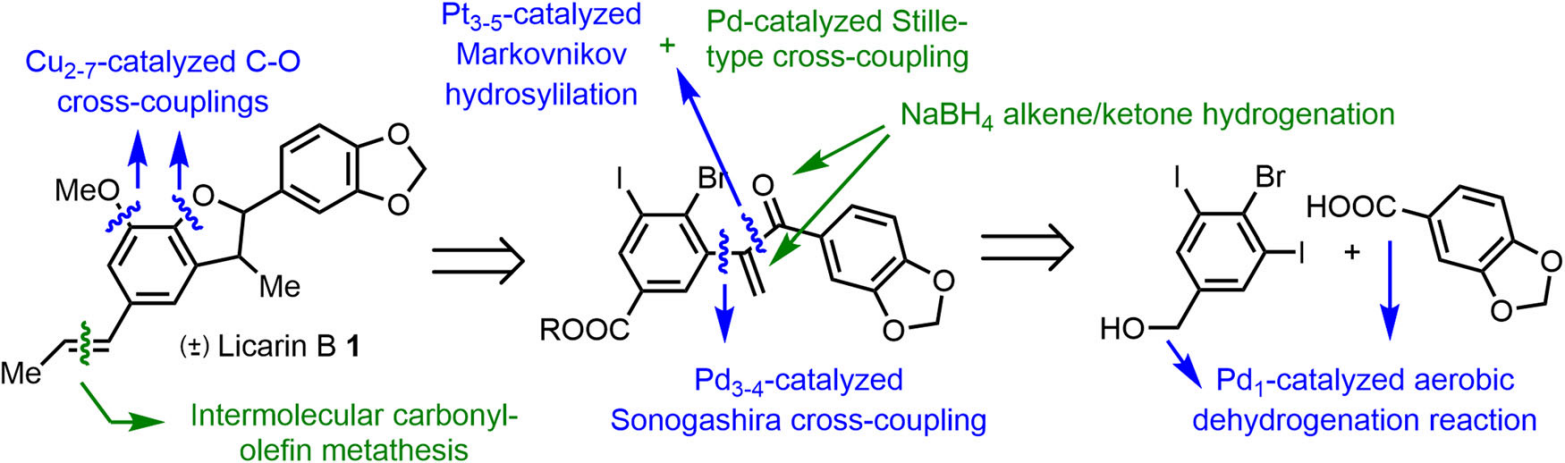
A total synthesis with individual and few-atoms metal catalysts. Retrosynthesis of (±)-Licarin B **1** based on individual and few-atoms metal catalyzed-reactions, in blue. The transformations in green correspond to other valuable reactions found during the study.

Figure 2 shows the retrosynthetic approach proposed here for (±)-**1**, also valid for other dehydrobenzofurane (neo)lignans. The synthetic route is based on diverse single and few-atoms metal catalyzed-reactions, including dehydrogenation^26^, hydrosilylation^27^ and cross-coupling reactions^28,29^ with Pd, Pt and Cu single atoms and metal cluster catalysts in solution, and without any canonical ligand (phosphines, carbenes,…). It must be noticed here again that the concept “SACs” often refer to single atoms supported/embedded onto solid supports, however, we will use here these SACs (and also metal clusters) in solution, named “individual metal atoms”, since the latter allows free interaction with large molecules and avoids the inherent diffusion limitations associated to solid catalysts. However, the solid-supported counterparts for the ultrasmall catalytic metal species used here are as active as the catalysts in solution, at least for the reported reactions^26–28^, thus leaving room to also perform the reactions under heterogeneous reaction conditions. Besides, our retrosynthesis contains a recently reported intermolecular carbonyl-olefin metathesis^30^ as the last step of the synthesis, since this transformation affords a high *trans* alkene selectivity either under homogenous or heterogeneous reaction conditions. Furthermore, the intermolecular carbonyl-olefin metathesis reaction has to our knowledge been barely tested in synthetic programs, much less in a late stage synthetic step^31^. We will also show here that, unexpectedly, the hydrogenation of an intermediate ketone with NaBH_4_, without any apparent catalytic metal, will promote preferentially the hydrogenation of an alkene rather than the conjugated ketone. This reaction has somehow been reported for LiAlH_4_^32^ but it is difficult to find for NaBH_4_, as far as we know^33^. We think that all these features make the synthesis of (±)-**1** described here of interest for catalytic and synthetic chemists.

## Results

### Synthesis of intermediate 14

**Fig. 3:**
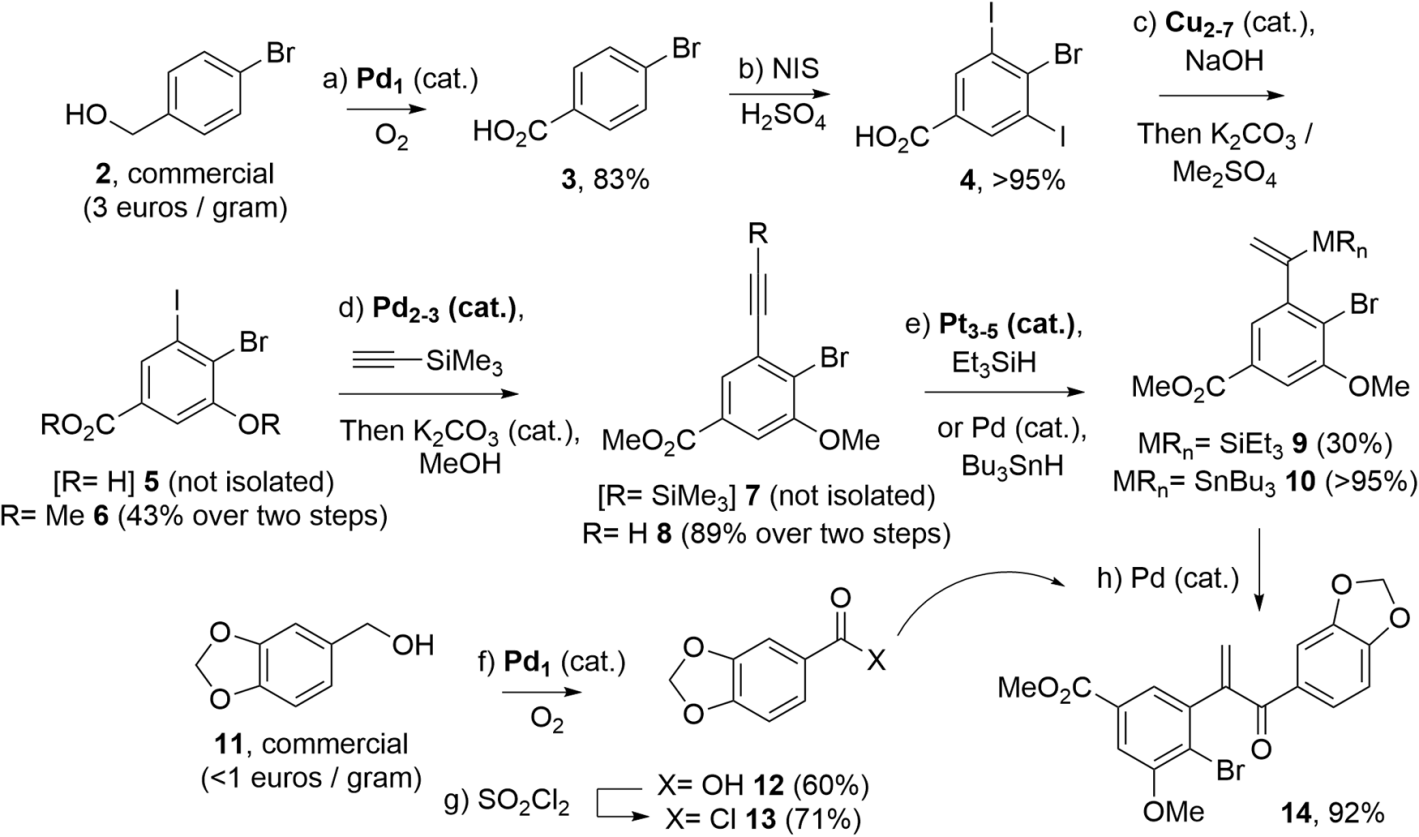
Results for the synthesis of (±)-Licarin B 1 (first part). Metal individual atoms and few-atoms metal clusters catalysts are marked in bold. All reactions were carried out with dried solvents and under nitrogen atmosphere otherwise indicated. Yields refer to isolated yields in all cases. Reaction conditions: (**a**) **2** (neat, 2 mmol), Pd_1_ [0.3 mol%, prepared from Pd(OAc)_2_], O_2_ (4 bar), 150 °C, 16 h; (**b**) **3** (49.8 mmol), H_2_SO_4(c)_ (0.3 M), *N*-iodosuccinimide (NIS, 128.3 mmol, 2.6 equiv.), room temperature, 16 h; (**c**) **4** (2.2 mmol), 120 mg (0.8 mmol, 0.4 equiv.), Cu_2-7_ (40 mol%, prepared from Cu_2_O), NaOH (12 mmol, 5.4 equiv.), H_2_O (10 ml), 85 °C, 16 h, then precipitation and treatment with K_2_CO_3_ (5.6 mmol, 2.2 equiv.), acetone (37 ml) and dimethyl sulfate (DMS, 5.5 mmol, 2.1 equiv.), 50 °C, 16 h; (**d**) **6** (0.4 mmol), trimethylsilylacetylene (0.8 mmol, 2 equiv.), Pd_2-3_ [0.1 mol%, prepared from Pd(OAc)_2_], KOAc (0.5 mmol, 1.25 equiv.), *N*-methyl pyrrolidone (NMP, 0.5 M), 150 °C, 20 h, then K_2_CO_3_ (20 mol%), MeOH (0.2 M); (**e**) **8** (0.16 mmol.), triethylsilane (0.19 mmol, 1.2 equiv.), Pt_3-5_ [0.7 mol%, prepared from Karstedt’s complex (Pt^0^-1,3-divinyl-1,1,3,3-tetramethyldisiloxane)], toluene (0.5 M), 110 °C, 16 h, or **8** (0.2 mmol), Pd(PPh_3_)_2_Cl_2_ (5 mol%), tetrabutylstannane (0.2 mmol, 1 equiv.), THF (0.5 M), 0 °C, 1.5 h; (**f**) As in (**a**); (**g**) **12** (1.1 mmol), SOCl_2_ (0.5 M), 80 °C, 3 h; (**h**) **10** (0.32 mmol), **13** (0.36 mmol, 1.1 equiv.), Pd(PPh_3_)_4_ (1 mol%), toluene (0.1 M), 110 °C, 16 h.

Figure 3 shows the first part of the (±)-Licarin B **1** synthesis, going from the commercially available and relatively cheap starting materials **2** and **11** to the coupled intermediate **14** (see Supplementary Methods and Supplementary Data 1 for NMR copies of the products). First, benzyl alcohol **2** is oxidized under aerobic conditions to the corresponding benzoic acid **3** in high yield (83%), without any solvent or additive, catalyzed Pd_1_ individual atoms formed in-situ after mild reduction of the Pd precursor [in this case Pd(OAc)_2_] with **2** (a complete substrate scope for this catalyst can be found in the previous study)^26^. The previous characterization of the catalytic Pd_1_ individual atoms, including a representative aberration-corrected high-angle annular dark field scanning-transmission electron microscopy (AC-HAADF-STEM) image and X-ray absorption near-edge structure (XANES) / extended X-ray absorption fine structure (EXAFS) spectra, can be found in the Supplementary Information (Fig. S1 and Table S1)^26^. The used catalyst was not characterized after reaction, however, this can be found in the precedent study^26^ (this applies to the single atom and cluster catalysts ahead). The isolated carboxylic acid **3** can now be easily di-iodinated in both *metha*-positions with *N*-iodosuccinimide (NIS) under acid conditions, to give the tri-halogen substituted intermediate **4** in >95% yield. A selective substitution of one of the I atoms on the ring, to give **5**, was accomplished with catalytic Cu clusters of 2 to 7 atoms (Cu_2-7_) in basic media. The characterization of the catalytic clusters here prepared was accomplished using absorption/emission ultraviolet-visible (UV-vis) spectrophotometric measurements and MALDI-TOF spectra [Fig. S2 top and Tables S2–S3, see the corresponding UV-vis and MALDI-TOF spectra of the previously reported Cu clusters in Fig. S2 bottom, for the sake of comparison, and a complete substrate scope for this catalyst can be found in the previous study]^29^. It is worth commenting here that the control over the mono-hydroxylation reaction, on just one single halogen site, is not easy and significant amounts of starting material **4** and di-hydroxylated compounds were concomitantly recovered after reaction. Nevertheless, a reasonable two-step yield of **6** (43%) was obtained after methylation of the two OH groups present in the molecule. The methylation of both OH groups after the Cu-catalyzed C-O cross-coupling reaction allows using inexpensive aqueous NaOH as a nucleophile and circumvents the possible degradation of a previously prepared ester group.

With compound **6** in hand, a Cu-free Sonogahira coupling between **6** and trimethylsilylacetylene, catalyzed by a 0.03 mol% of two- and three-atoms Pd clusters (Pd_2-3_), was carried out [again, the characterization of the catalytic clusters was carried out with absorption/emission UV-vis and MALDI-TOF measurements (Fig. S3 top and Tables S2, S4) and compared with the previously reported Pd clusters (Fig. S3 bottom), and a complete substrate scope for this catalyst can be found in previous studies]^28,34^. The Pd_2-3_ clusters were prepared after mild reduction of Pd(OAc)_2_ in aqueous (1 wt%) *N*-methylpyrrolidone (NMP), stored in solution and added to the reaction mixture [0.1 mol%, see absorption/emission UV-vis spectrophotometry and matrix-assisted laser desorption/ionization coupled to time-of-flight (MALDI-TOF) mass spectrometry spectra in Fig. S3]^28^. The corresponding Sonogashira product **7** was obtanied in low yield (17%), lower to that obtanied by a conventional procedure with PdCl_2_(PPh_3_)_2_ and CuI co-catalysts (>95%, see Supplementary Methods, Fig. S4). However, it is noteworthy that the amount of Pd is two orders of magnitude lower for clusters and Cu is not used (turnover number, TON = 566 for Pd_2-3_ vs 20 for the organometallic complex). A one-pot base-catalyzed desilylation reaction gives the terminal phenylacetylene derivative **8** in 89% yield after two steps without isolation of **7**.

The C-Br bond in **8** may, in principle, allow to construct the dehydrobenzofurane core of **1** using a C-O cross-coupling reaction with Cu clusters as a catalyst and piperonyl alcohol as a nucleophile, such as in the synthesis of **5** (Fig. S5). However, the intermolecular C-O cross-coupling reaction proved fruitless, in accordance with the lower reactivity of C-Br respect to C-I bonds and the high steric hindrance around the C-Br bond. Thus, we turned our attention to piperaldehyde as an electrophile, instead of piperonyl alcohol as a nucleophile, and to activate the α-position of the terminal alkyne in **8**. Under reported conditions with Et_2_AlH as a hydroaluminating agent and Ti(O^i^Pr)_4_ as an activator of the aldehyde electrophile^35^, any coupled product was not observed (Fig. S6). Thus, we decided to pre-activate the alkyne α-position with a good transmetallable or leaving group, to then carry out the coupling reaction with the piperonyl moiety.

Pt clusters between 3 and 5 atoms (Pt_3-5_) were prepared by heating the Karstedt’s complex (Pt^0^-1,3-divinyl-1,1,3,3-tetramethyldisiloxane) in toluene solution, and characterized by absorption/emission UV-vis and MALDI-TOF measurements (Fig. S7 top and Tables S2, S5), comparing with the previously reported Pt clusters (Fig. S7 bottom, with a representative AC-HAADF-STEM image, the emission UV-vis spectrum and the high-resolution mass spectrum in an ORBITRAP instrument)^36,37^. They were applied as a catalyst for the regioselective Markovnikov hydrosilylation of the terminal alkyne in **8** (a complete substrate scope for this catalyst can be found in the previous study)^27,36^. However, the yield of product **9** was low (30%). Besides, the attempted coupling of **9** with piperonaldehyde, catalyzed by a phosphazene compound^38^, failed (Fig. S8). Thus, we tested a related Pd-catalyzed procedure with tetrabutylstannane^39^, to give product **10** in >95% yield, having the required Markovnikov regioselectivity. With **10** in hand, we tested different reactions to couple piperaldehyde and thus leave a free alcohol group for the later cyclization reaction (Fig. S9), however, none of the reported conditions^39–41^ worked in our hands.

At this point, we envisioned a different coupling reaction to achieve the diaryl substituted *gem*-conjugated ketone **14** instead of the alcohol, based on the use of acyl chloride **13** as a coupling partner. The latter was obtained in two steps, first performing the Pd_1_-catalyzed aerobic oxidation of piperonyl alcohol **11**, under the same reaction conditions than for the starting material **2** [notice that reported Pinnick oxidation conditions from piperonal^42^ gave, in our hands, lower conversion than the Pd_1_-catalyzed aerobic oxidation (Fig. S10)] and subsequent chlorination of acid **12**. Now, the synthesis of **14** from **10** and **13** was attempted by a Pd-catalyzed Stille-type coupling reaction catalyzed by Pd(PPh_3_)_4_ under reported reaction conditions^43^, which gave **14** in 92% yield. The use of a Pd-catalyzed reaction is an alternative methodology to the use of typical CrCl_2_-mediated Nozaki-Hiyama-Kishi reaction conditions^44^, which nevertheless failed here, after forming the corresponding vinyl iodide from **10** (Fig. S11). Unfortunately, the use of Pd_2-3_ clusters as a catalyst for the Stille-type coupling did not give the desired product (Fig. S12)^28^.

### Completion of the synthesis

**Fig. 4:**
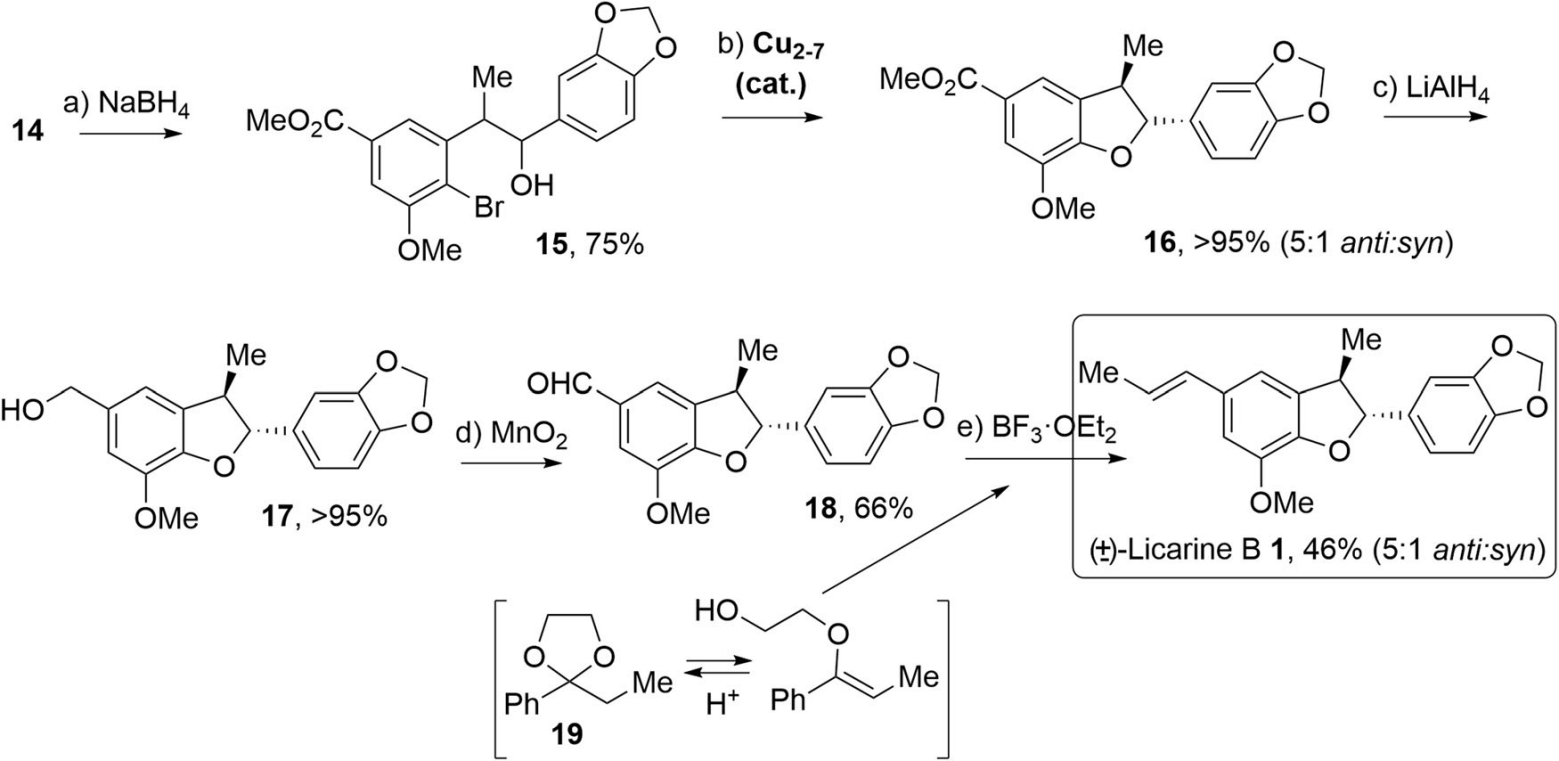
Completion of the synthesis of (±)-Licarin B 1. Metal individual atoms and few-atoms metal clusters catalysts are marked in bold. All reactions were carried out with dried solvents and under nitrogen atmosphere otherwise indicated. Yields refer to isolated yields in all cases. Reaction conditions: (**a**) **14** (0.25 mmol), NaBH_4_ (0.6 mmol, 2.4 equiv.), THF:MeOH 1:1 (0.1 M), room temperature, 16 h; (**b**) **15** (0.014 mmol), Cu_2-7_ (10 mol%, prepared from CuI), Cs_2_CO_3_ (0.02 mmol, 1.5 equiv.), DMF (0.05 M), 130 °C, 16 h; (**c**) **16** (0.05 mmol), LiAlH_4_ (0.20 mmol, 4 equiv.), THF (0.1 M), room temperature, 16 h; (**d**) **17** (0.044 mmol), activated MnO_2_ (0.27 mmol, 6 equiv.), DCM (0.5 M), room temperature, 16 h; (**e**) **18** (0.012 mmol), **19** (0.036 mmol, 3 equiv.), BF_3_·OEt_2_ (0.012 mmol, 1 equiv.), DCE (0.03 M), 70 °C, 40 h.

Figure 4 shows the completion of the synthesis (see Supplementary Methods and Supplementary Data 1 for NMR copies of the products). The coupled intermediate **14** was treated with an excess (2.4 equivalents) of NaBH_4_, in order to hydrogenate the ketone. However, for our surprise, the alkene functionality was also hydrogenated, even faster than the ketone, thus both the methyl and the alcohol groups were formed at once, to give **15** in 75% yield. Indeed, the ketone intermediate could be isolated and characterized (Fig. S13). Inductively coupled plasma-optical emission spectroscopy (ICP-OES) analyses showed that the metal content in the reaction mixture is extremely low for all metals analysed, except for Sn, Si and B, and, of course, Na (Table S6, sample A). In order to check if some Sn traces, remaining in reactant **14** after purification of the reaction with **10**, were catalyzing the hydrogenation of the alkene with NaBH_4_, a new batch of **14** with higher amounts of Sn (Table S6, sample B) was tested, however, the hydrogenation of the alkene was slower than before, while the ketone was hydrogenated somewhat faster. Thus, we have to preliminary conclude here that NaBH_4_ is able to hydrogenate the alkene group in **14** by its own, which constitutes a practical reaction (hydrogenation of alkenes with NaBH_4_) with any uncatalyzed precedent, as far as we know^45^. This methodology adds to related synthetic strategies, such as the circumvention of carbonyl group reduction challenges under Luche reduction^46^. Notice here that racemic 1,2-diarylethanols can be easily converted to enantiomerically pure compounds by dynamic kinetic resolution^47^, thus enabling the access to the corresponding enantiomeric forms of (±)-**1**.

The C-O intramolecular coupling in **15** was then attempted with Cu_2-7_ clusters as catalysts, as previously carried out for intermediate **5**. Despite a C-Br bond is more difficult to activate than a C-I bond^48^, we envisioned that the formation of a very stable dehydrobenzofuran core would entropically help to the reaction to proceed, moreover with the *O*-nucleophile activated by a base. In contrast to the synthesis of **5**, we avoided aqueous conditions to not generate hydroxyls in the medium, thus NMP was used as the solvent. Under these reaction conditions, the Cu_2-7_ clusters catalyzed the reaction quantitatively, to obtain **16** in >95% yield. Hydrogenation of the ester to alcohol **17** and dehydrogenation of the latter to aldehyde **18**, under classical reaction conditions (with LiAlH_4_ and activated MnO_2_, respectively) gave intermediate **18** in >60% yield after two steps.

The final step consists in the transformation of the aldehyde in **18** into a *trans* ethylene group. This transformation is not straightforward by classical methodologies since Wittig-type reactions either lead to the *cis* alkene product or require the activation of the alkene with electron withdrawing groups to have a reasonable *trans* selectivity. Thus, we envisioned the use of a recently reported by us^30^ acid-catalyzed intermolecular carbonyl-olefin metathesis reaction, with high *trans* selectivity and which does not require activated alkenes. For that, we prepared ketal **19** which, under the acidic reaction conditions, stays in equilibrium with the corresponding vinyl ether form, to engage with aldehyde **18** in an intermolecular carbonyl-olefin metathesis reaction and give the desired (±)-Licarin B **1** in 46% yield. The ketal without phenyl group was also prepared^49^ and tested (Fig. S14), however, did not show the desired reactivity of **19**. To our knowledge, examples of intermolecular carbonyl-olefin metathesis during late stages of a complex organic synthesis are extremely rare^31^. In order to check the formation of the *trans* alkene isomer, we additionally performed the synthesis of (±)-**1** by a Takai reaction^50^, which is arguably the most reliable methodology to get a *trans* ethylene product from an aldehyde, however, at expenses of not fulfilling any sustainable principle for modern chemistry, since employs stoichiometric amounts of anhydrous CrCl_2_^51^. For that, we prepared ethyl diiodide by a recently reported zeolite-catalyzed halogen exchange reaction^52^, avoiding the classical hydrazine-based methodology^53^. The Takai reaction with **18** gave (±)-**1** in very high yield (93%) and a *trans/cis* ratio >20:1 (Fig. S15), similar not only to the product of the carbonyl-olefin metathesis reaction but also to a commercial sample of enantiomerically pure **1**. Comparison of the synthesized (±)-Licarin B **1** with a commercial sample by Fourier transform infrared spectra (FT-IR), gas chromatography (GC), UV/vis spectrophotometry, and ^1^H and ^13^C nuclear magnetic resonance (NMR) spectra (Figs. S16–S20) validate our synthetic route, and shows that the regioselectivity of the as-synthesized (±)-**1** is enriched in the *anti* regioisomer (ca. 5:1), as in natural Licarin B **1** (pure *anti* regioisomer). We infer that the enrichment in the natural regioisomer occurs by a favourable conformation during the cyclization reaction. Notice that acetone and DCM solvent traces are contained in both our synthesized **1** and the commercial sample, with FT-IR peaks at 1650 and 700 cm^−1^, and ^1^H NMR signals at 5.3 and 2.17 ppm, respectively, and that the ^13^C NMR signal at 125.5 ppm may correspond to the small amount of *cis* alkene product formed during the Takai reaction.

## Conclusions

The synthesis of (±)-Licarin B **1** has been accomplished in 11 linear steps with 6 of these steps catalyzed by individual metal atoms and few-atom metal clusters in solution. The overall yield is 13.1%, higher than any of the previous synthesis reported >30 years ago. The synthetic route could be easily diverted to achieve other members of the neolignan family (see Fig. 1) and also to obtain the diastereomerically pure isomers. Other remarkable features found here are a late stage intermolecular carbonyl-olefin metathesis and an apparently metal-free hydrogenation of an alkene with NaBH_4_, faster than the hydrogenation of a ketone. These results, overall, can open new ways in the utilization of individual and few-atoms metal catalysts for total synthesis and new synthetic pathways for the synthesis of dehydrobenzofurane neolignans.

## Methods

### General

Reagents and solvents were obtained from commercial sources and were used without further purification otherwise indicated. The commercial sample of enantiomerically pure Licarin B **1** shows a 97% purity grade. Glassware was dried in an oven at 175 °C before use. Reactions were performed in round-bottomed flasks or 2.0 ml vials closed with a steel cap, equipped with a magnetic stirrer and having a rubber septum part to sample out. A nitrogen atmosphere was set for all reactions, otherwise indicated. Products were characterized by gas chromatography-mass spectrometry (GC-MS), high resolution electrospray ionization-mass spectrometry (ESI-MS), ^1^H- and ^13^C- nuclear magnetic resonance NMR, and distortionless enhancement by polarization transfer (DEPT). GC analyses were performed in an instrument equipped with a 25 m capillary column of 5% phenylmethylsilicone. Nitrobenzene or *N*-dodecane were used as an external standard. GC-MS analyses were performed on a spectrometer equipped with the same column as the GC and operated under the same conditions. ^1^H, ^13^C and DEPT measurements were recorded in a 300 or 400 MHz instrument using CDCl_3_ (containing TMS as an internal standard) and DMSO-*d6* as a solvent. The metal content of the catalyst solutions and of the reaction mixture with NaBH_4_ was determined by inductively coupled plasma-optical emission spectroscopy (ICP-OES) after disaggregation of the mixture in acidic water mixtures: typically, 0.5 ml of the mixture were treated with 5 ml of *aqua regia* for 5 min, diluting with 50 ml of bi-distilled water and analyzing, after quantification by comparison of the response with a calibration plot. Absorption and emission ultraviolet-visible spectroscopy measurements were performed on the same samples than that for mass determination, using an UV/Vis (UV0811M209, Varian) and a LP S-220B spectrophotometer (Photon Technology International, equipped with 75 W Xe lamp), respectively.

### Microscopy measurements

Pd_1_ and Pt_3-5_ species were characterized by electron microscopy after depositing one drop of the synthesized clusters in solution (or trapped in charcoal) onto holey-carbon coated Cu grids. After their preparation, samples were conserved under vacuum conditions. Scanning-transmission electron microscopy studies, using high-angle annular dark-field (HAADF- STEM), were performed on a FEI Titan Themis 60–300 double aberration corrected microscope operated at 300 kV. To obtain images with good quality, the beam current and image acquisition time were optimized according to the stability of the sample under the beam.

### X-Ray Absorption Structure (XAS) techniques

X-ray absorption near edge structure (XANES) and extended X-ray absorption fine structure (EXAFS) measurements for Pd_1_ were carried out on CLAESS beamline at ALBA Synchrotron Light Source, Barcelona, Spain. The synchrotron light coming from the multipole wiggler has been first vertically collimated, then monochromatized using two pairs of liquid nitrogen cooled Si(311) crystals and finally focused on the sample position down to ~500 × 500 μm^2^. Rh stripe coating on the two optical mirrors guarantees the higher harmonics rejection. The Pd in solution sample was prepared in a 10 ml glass vial equipped with a stirring bar, adding benzyl alcohol (1.96 mmol, 200 µl) and 0.08% mol of Pd(OAc)_2_ (0.4 mg). The vial was closed with a septum equipped with a manometer and charged with air. Then the mixture was placed in a pre-heated metal heating plate at 150 °C and stirred at 450 rpm for 30 min. The XAS data were obtained in fluorescence mode at the Pd K-edge (24.350 keV) for our samples and transmission mode for the standards. Energy scale was calibrated by measuring a Pd foil sample. The local structure of the sample was refined using the EXAFS signal in the k range 3:10 Å^−1^.

### Orbitrap measurements

The mass of the Pt_3-5_ clusters were measured in a flow injection-HRMS consisted of an injection and pump systems and a single mass spectrometer Orbitrap Thermo Fisher Scientific (Exactive™) using an electrospray interface (ESI) (HESI-II, Thermo Fisher Scientific) in positive or negative mode. The sample (10 μl) was injected into the flow-injection solvent consisting of an aqueous solution of 0.1% formic acid and methanol (1:1). The mass spectra were acquired employing two alternating acquisition functions: full MS, ESI + , without fragmentation and all-ion fragmentation (AIF), ESI + , with fragmentation. The mass range was 150.0–1500.0 *m/z*.

### Matrix-assisted laser desorption/ionization coupled to time-of-flight (MALDI-TOF) mass spectrometry

MALDI-TO measurements for the Pd_2-3_ clusters were performed on the solution in DMF or NMP, diluted in 1 ml of acetonitrile (1 μL of the final solution was spotted onto the MALDI plate). After the droplets were air-dried at room temperature, 0.5 μL of matrix (5 mg/mL CHCA (Bruker) in 0.1% TFA-ACN/H_2_O (7:3, v/v) was added and allowed to air-dry at room temperature. The resulting mixtures were analyzed in a 5800 MALDI TOFTOF (ABSciex) in positive reflectron mode (3000 shots every position) in a mass range of 150–1500 m/z. Previously, the plate and the acquisition method were calibrated with 1 µL the TOFTOF calibration mixture (ABSciex), in 13 positions.

### Synthesis of the individual and few-atom metal catalysts

The synthesis of Pd_1_ individual atoms was performed from Pd(OAc)_2_ following a reported procedure^26^. In brief, the Pd salt was heated in the benzyl alcohol under air, to in-situ form the Pd_1_ single atoms after mild reduction with the alcohol. The synthesis of Cu_2-7_^29^, Pd_2-3_^28^ and Pt_3-5_^36^ clusters was also performed by reported procedures, briefly, by mild reduction in amide solvents (DMF or NMP) while heating at 120–150 °C for a few minutes. For Pd_2-3_ clusters, a 1 wt% of water was added to stabilize the clusters.

### Reaction procedures

#### Synthesis of 4-bromobenzoic acid 3

In a 10-mililiter sealed reactor, 365.6 mg (1.96 mmol, 1 equiv.) of 4-bromo benzyl alcohol (**2**) were heated at 150 °C along with 1.32 mg (0.006 mmol, 0.003 equiv.) of Pd(OAc)_2_ under an O_2_ atmosphere of 4 atm. After 4 h, the temperature was cooled down and the reaction progress was checked by ^1^H NMR and GC. A distribution of three products was obtained in a proportion 1:1:1. As there was a 49% conversion, the reactor was charged with 2 atm of O_2_ and heated for 16 h. After that time, it was obtained full conversion, with an 83% yield of the target benzoic acid after hydrolysis of the ester formed.

#### Synthesis of 4-bromo-3,5-diiodobenzoic acid 4

In a one-neck round bottom flask, 9.96 g (49.75 mmol, 1 equiv.) of **3** were dissolved in 184 mL of H_2_SO_4_(c) and, then, the mixture was cooled down to 0 °C. 28.7 g (128.35 mmol, 2.6 equiv.) of *N*-iodosuccinimide (NIS) were added portionwise. The reaction was stirred overnight at room temperature. A mixture of 250 mL water/ice was added slowly and stirred for 30 min. Next, 7.4 g of Na_2_S_2_O_3_ dissolved in 74 mL of deionized water were added. After 2 h of mixing, the solid was filtered by vacuum and was treated with deionized water until fully disappearance of turbidity in the liquid phase. Compound **3** was isolated as a coral powder (22.4 g, 99% yield). ^1^H NMR (401 MHz, DMSO) δ 8.32 (s, 2H). ^13^C NMR (75 MHz, DMSO) δ 164.27, 140.52, 139.92, 132.25, 101.90. IR υ max : 2513, 1684, 1521, 1361, 1280 cm^−1^.

#### Synthesis of 4-bromo-3-hydroxy-5-iodobenzoic acid 5

In a 25-mililiter flask, 1 g (2.21 mmol, 1 equiv.) of **4**, 120 mg (0.84 mmol, 0.4 equiv.) of Cu_2_O and 475 mg (11.88 mmol, 5.4 equiv.) of NaOH (previously prepared in 10 mL of deionized water) were mixed in a preheated oil bath at 85 °C during 16 h. Then, the pH was adjusted at 2 and the content was poured in a 10-mililiter mixture of water/ice. The solid was filtered by vacuum. 880 mg of a mixture of **5** and **4** (1:1.3) were isolated as a light brown solid. ^1^H NMR (401 MHz, DMSO) δ 13.55 (s, 1H), 11.05 (s, 1H), 7.84 (d, *J* = 1.6 Hz, 1H), 7.47 (d, *J* = 1.6 Hz, 1H). IR υ max : 3204, 2808, 1686, 1570, 1405, 1283, 1243, 1090 cm^−1^.

#### Synthesis of methyl 4-bromo-3-iodo-5-methoxybenzoate 6

880 mg of a mixture of **4** and **5** (1.3:1) were dissolved in a 25-mililiter flask with 37 mL of acetone along with 770 mg (5.57 mmol, 2.17 equiv.) of K_2_CO_3_. Then, 530 µL (5.53 mmol, 2.15 equiv.) of DMS (dimethyl sulfate) were added and the stirring was kept overnight at 50 °C. Next, the mixture was filtered and the acetone evaporate. The crude was re-dissolved in AcOEt and NaHCO_3_ (sat.) was added. Three extractions with AcOEt were carried out and the organic phase was washed with NaCl (sat), dried with MgSO_4_, filtered and the solvent was removed by vacuum. The crude was purify by chromatographic column, using hexane:DCM (85:15) as an eluent. 415 mg of **6** as a white powder were isolated [99% yield referred to the initial proportion of **5**:**4** (1:1.3)]. ^1^H NMR (401 MHz, CDCl_3_) δ 8.14 (d, *J* = 1.8 Hz, 1H), 7.49 (d, *J* = 1.8 Hz, 1H), 3.93 (s, 3H), 3.92 (s, 3H). ^13^C NMR (101 MHz, CDCl_3_) δ 165.44, 156.72, 133.13, 131.30, 125.21, 111.69, 102.88, 57.01, 52.75. IR υ max : 1703, 1556, 1390, 1280, 1241, 1190, 1123, 1055, 1021 cm^-1^.

#### Synthesis of methyl 4-bromo-3-methoxy-5-((trimethylsilyl)ethynyl)benzoate 7

*Method A (Pd*_*2-3*_ *clusters)*: In a vial, 10 mg (0.027 mmol, 1 equiv.) of **6** were dissolved in 60 μL of NMP along with 8 μL (0.053 mmol, 2 equiv.) of trimethylsilylacetylene, 3.8 mg (0.039 mmol, 1.4 equiv.) of KOAc and the appropriated quantity of Pd(Ac)_2_ to get 300 ppm of Pd in the mixture. The mixture was stirred 20 h at 130 °C. Then, an aliquot was taken and analyzed by GC. A conversion of 45% took place, with a selectivity towards the desired product of 37% (the dehalogenated byproduct complted the mass balance). *Method B [Pd(PPh*_*3*_*)*_*2*_*Cl*_*2*_ *and CuI]:* In a 10-mililiter flask, 149 mg (0.40 mmol, 1 equiv.) of **6**, 15 mg (0.081 mmol, 0.2 equiv.) of CuI, 180 μL (1.21 mmol, 3 equiv.) of NEt_3_ and 14 mg of Pd(PPh_3_)_2_Cl_2_ were dissolved in 1.8 mL of dry 1,4-dioxane under inert atmosphere. Then, 80 μL (0.53 mmol, 1.3 equiv.) of trimethylsilylacetylene were added and the mixture was stirred at 80 °C for 16 h. After cooling the reaction, 15 mL of AcOEt were added and the organic phase washed twice with HCl 0.5 M, once with NaCl (sat), dried with anhydrous MgSO_4_, filtered and the solvent removed by vacuum. 130 mg of **7** as a brownish powder were obtained (94% yield). ^1^H NMR (401 MHz, CDCl_3_) δ 7.78 (d, *J* = 1.9 Hz, 1H), 7.47 (d, *J* = 1.9 Hz, 1H), 3.95 (s, 3H), 3.92 (s, 3H), 0.28 (s, 9H). ^13^C NMR (101 MHz, CDCl_3_) δ 166.05, 156.49, 129.96, 127.14, 126.84, 120.85, 112.01, 102.33, 100.95, 56.80, 52.62, −0.09. HRMS: m/z calculated for C_14_H_18_BrO_3_Si (M + H^+^): 341.0208; found: 341.0187. IR υ max : 1712, 1566, 1403, 1239, 1217 cm^−1^.

#### Synthesis of methyl 4-bromo-3-ethynyl-5-methoxybenzoate 8

In a 25-mililiter flask, 130 mg (0.38 mmol, 1 equiv.) of **7** and 15 mg (0.08 mmol, 0.2 equiv.) of anhydrous K_2_CO_3_ were dissolved in 2 mL of dry MeOH. The mixture was stirred for 3 h, diluted with 5 mL of AcOEt and filtered. Then, the crude was purify by chromatographic column using hexane:AcOEt as an eluent 95:5. 101 mg of **8** were isolated as a brownish powder (99% yield). ^1^H NMR (300 MHz, CDCl_3_) δ 7.74 (d, *J* = 1.9 Hz, 1H), 7.44 (d, *J* = 1.9 Hz, 1H), 3.89 (s, 3H), 3.86 (s, 3H), 3.35 (s, 1H). ^13^C NMR (75 MHz, CDCl_3_) δ 165.76, 156.40, 130.02, 127.01, 126.02, 120.72, 112.31, 82.68, 81.24, 56.88, 56.68. HRMS: m/z calculated for C_11_H_10_BrO_3_ (M + H^+^): 268.9813; found: 268.9795. IR υ max : 3253, 1706, 1566, 1400, 1324, 1238, 1214 cm^-1^.

#### Synthesis of methyl 4-bromo-3-methoxy-5-(1-(triethylsilyl)vinyl)benzoate 9

In a vial, 42 mg (0.16 mmol, 1 equiv.) of **8** were dissolved in 210 μL of toluene along with 30 μL (0.19 mmol, 1.2 equiv.) of HSiEt_3_ and stirred at 110 °C. Then, 35 mg (0.001 mmol, 0.007 equiv.) of Karsdest’s catalyst were dissolved in 100 μL of toluene and added over the previous mixture. The mixture was stirred for 16 h. Next, the solvent was removed by vacuum and the crude purify by chromatographic column, using hexane:AcOEt as an eluent. 20 mg of **9** were isolated as a colorless oil (33% yield). ^1^H NMR (401 MHz, CDCl_3_) δ 7.38 (d, *J* = 2.0 Hz, 1H), 7.29 (d, *J* = 1.9 Hz, 1H), 5.77–5.72 (m, 2H), 3.96 (s, 3H), 3.95 (s, 3H), 1.04–0.81 (m, 9H), 0.71–0.60 (m, 6H).

#### Synthesis of methyl 4-bromo-3-methoxy-5-(1-(tributylstannyl)vinyl)benzoate 10

In a 10-mililiter flask, 52 mg (0.19 mmol, 1 equiv.) of **8** and 6.8 mg (0.01 mmol, 0.05 equiv.) of Pd(PPh_3_)_2_Cl_2_ were dissolved in 700 μL of dry THF under inert atmosphere. The temperature was lowered to 0 °C and 60 μL (0.20 mmol, 1.05 equiv.) of HSnBu_3_ were added. After 1.5 h of reaction, the crude was purify by chromatographic column, using hexane:AcOEt 98:2 as an eluent. **10** was obtained as a colorless oil (104 mg, 96% yield). ^1^H NMR (401 MHz, CDCl_3_) δ 7.36 (d, *J* = 1.9 Hz, 1H), 7.32 (d, *J* = 1.9 Hz, 1H), 5.79 (d, *J* = 2.6 Hz, 1H), 5.52 (d, *J* = 2.6, 1H), 3.95 (s, 3H), 3.91 (s, 3H), 1.51–1.38 (m, 6H), 1.26 (h, J = 7.3 Hz, 6H), 0.97–0.90 (m, 6H), 0.85 (t, J = 7.3 Hz, 9H). ^13^C NMR (101 MHz, CDCl_3_) δ 13 C NMR (101 MHz, CDCl3) δ 166.86, 156.64, 155.82, 150.34, 129.82, 128.70, 121.63, 116.45, 109.30, 56.70, 52.43, 29.84, 29.34, 29.22, 29.12, 29.02, 28.92, 27.71, 27.55, 27.42, 27.13, 14.45, 13.86, 13.77, 13.01, 12.00, 11.37, 11.35, 10.84. HRMS: m/z calculated for C_23_H_38_BrO_3_Sn (M + H^+^): 561.1026; found: 561.0995. IR υ max :2921, 2359, 1723, 1570, 1395, 1318, 1235 cm^−1^.

#### Synthesis of benzo[*d*][1,3]dioxole-5-carboxylic acid 12

*Method A (Pd*_*1*_ *single atom):* In a 10-mililiter sealed reactor, 298 mg (1.96 mmol, 1 equiv.) of piperonyl alcohol (**11**) were heated along with 1.32 mg (0.006 mmol, 0.003 equiv.) of Pd(AcO)_2_ at 150 °C under an O_2_ atmosphere of 4 atm, overnight. After that, the crude was analyzed by ^1^H NMR, obtaining a 4:1mixture of piperonal and **12**, with a conversion of 86%. *Method B (Pinnick oxidation):* In a 25-mililiter flask, 298.3 mg (2 mmol, 1 equiv.) of piperonal were dissolved along with 138.3 mg (0.8 mmol, 0.4 equiv., 70 wt%) of NaH_2_PO_4_ and 2 mL of H_2_O_2_ (21.2 mmol, 10.6 equiv., 30 wt%) in 12 mL of a mixture MeCN:H_2_O 5:1. The temperature was lowered to 0 °C and 368 mg (4 mmol, 2 equiv.) of NaClO_2_ dissolved in deionized water were added dropwise. The mixture was stirring at room temperature overnight. 20 mL of HCl 2 M were added and extracted three times with AcOEt. Next, three extractions with NaOH 2 M were carried out and acidified with HCl(c), until pH 2, approximately. Finally, three additional extractions with AcOEt were done. This organic phase was washed twice with NaCl (sat), dried over anhydrous MgSO_4_, filtered and the solvent removed by vacuum. 220 mg of **12** were isolated as a slightly brown powder (68% yield). ^1^H NMR (300 MHz, DMSO) δ 12.70 (s, 1H), 7.54 (dd, *J* = 8.1, 1.7 Hz, 1H), 7.36 (d, *J* = 1.7 Hz, 1H), 7.00 (d, *J* = 8.1 Hz, 1H), 6.12 (s, 2H). ^13^C NMR (75 MHz, DMSO) δ 166.61, 151.12, 147.47, 124.96, 124.65, 108.78, 108.07, 101.93. HRMS: m/z calculated for C_8_H_7_O_4_ (M + H^+^): 167.0344; found: 167.0331. IR υ max : 2921, 2518, 1661, 1448, 1292, 1257 cm^−1^.

#### Synthesis of benzo[*d*][1,3]dioxole-5-carbonyl chloride 13

In a 25-mililiter flask, 183 mg (1.10 mmol, 1 equiv.) of **12** were dissolved in 2 mL of SOCl_2_ under inert atmosphere and refluxed for 3 h. Then, SOCl_2_ was removed by vacuum and the brown solid was re-dissolved in DCM and filtered through Al_2_O_3_. After evaporating the solvent, compound **13** was obtained as a light brown powder (145 mg, 71% yield). ^1^H NMR (401 MHz, CDCl_3_) δ 7.80 (dd, *J* = 8.3, 1.9 Hz, 1H), 7.52 (d, *J* = 1.9 Hz, 1H), 6.89 (d, *J* = 8.3 Hz, 1H), 6.11 (s, 2H). ^13^C NMR (75 MHz, CDCl_3_) δ 177.11, 154.05, 148.50, 136.36, 129.04, 110.78, 108.45, 102.71. IR υ max : 1738, 1503, 1601, 1261, cm^−1^.

#### Synthesis of methyl 3-(3-(benzo[*d*][1,3]dioxol-5-yl)-3-oxoprop-1-en-2-yl)-4-bromo-5-methoxybenzoate 14

*Method A (Pd*_*2-3*_ *clusters):* In two vials, 16 mg (0.03 mmol, 1 equiv.) of **10** were dissolved along with 0.38 mg (3.3·10^−4 ^mmol, 0.01 equiv.) of Pd(PPh_3_)_4_ and 5.6 mg (0.03 mmol, 1 equiv.) of **13** in 100 µL of DMF dry. One of the vials also contained 11.7 mg (0.033 mmol, 1.1 equiv.) of Cs_2_CO_3_. The reactions were heated at 120 °C for 16 h. Then, they were quenched with deionized water and extracted thrice with AcOEt. The organic phase was washed with NaCl (sat), dried over MgSO_4_, filtered and the solvent removed by vacuum. The remaining mixture was analyzed by GC-MS. *Method B [Pd(PPh*_*3*_*)*_*4*_*]:* In a Schlenk tube, 181 mg (0.32 mmol, 1 equiv.) of **10** were dissolved along with 66 mg (0.36 mmol, 1.1 equiv.) of **13** and 4.3 mg of Pd(PPh_3_)_4_ in 4.5 mL of dry toluene under inert atmosphere. The mixture was mixed in a preheated sand bath at 110 °C for 16 h. The crude was directly purify by chromatographic column using a mixture of hexane:AcOEt from 87.5:12.5 to 75:25 as an eluent. 125 mg of **14** were isolated as a colorless oil (92% yield). ^1^H NMR (300 MHz, CDCl_3_) δ 7.68 (d, *J* = 1.9 Hz, 1H), 7.60 (dd, *J* = 8.1, 1.7 Hz, 1H), 7.55 (d, *J* = 1.9 Hz, 1H), 7.46 (d, *J* = 1.9 Hz, 1H), 6.86 (d, *J* = 8.1 Hz, 1H), 6.08 (d, *J* = 0.8 Hz, 1H), 6.06 (s, 2H), 6.00 (d, *J* = 0.8 Hz, 1H) 3.53 (s, 3H), 3.34 (s, 3H). ^13^C NMR (101 MHz, CDCl_3_) δ 193.16, 166.27, 156.07, 151.62, 148.46, 147.85, 141.87, 131.31, 130.32, 128.30, 126.60, 124.66, 117.80, 111.93, 109.96, 107.76, 101.85, 56.68, 52.46. HRMS: m/z calculated for C_19_H_16_BrO_6_ (M + H^+^): 419.0130; found: 419.0113. IR υ max : 2359, 2341, 1718, 1438, 1239 cm^−1^.

#### Synthesis of methyl 3-(1-(benzo[*d*][1,3]dioxol-5-yl)-1-hydroxypropan-2-yl)-4-bromo-5-methoxybenzoate 15

In a 10-mililiter flask, 106 mg (0.25 mmol, 1 equiv.) of **14** and 24 mg (2.4 equiv.) of NaBH_4_ were dissolved in a 2-mililiter mixture of dry THF:MeOH 1:1. The stirring was kept overnight at room temperature. Next, 15 mL of deionized water were added and extracted thrice with DCM. The organic phase was washed with NaCl (sat), dried with anhydrous MgSO_4_, filtered and the solvent was removed by vacuum. 80 mg of **15** were isolated as a white powder (75% yield). ^1^H NMR (401 MHz, CDCl_3_) δ 7.66 (dd, *J* = 6.3, 1.9 Hz, 1H), 7.44 (d, *J* = 1.8 Hz, 1H), 6.95 (t, *J* = 1.7 Hz, 1H), 6.01 - 5.96 (m, 2H), 4.75 (d, *J* = 7.0 Hz, 1H), 3.97 (d, *J* = 0.9 Hz, 3H), 3.94 (s, 1H), 3.85 - 3.74 (m, 1H), 1.02 (d, *J* = 7.0 Hz, 3H). ^13^C NMR (101 MHz, CDCl_3_) δ 166.73, 156.26, 148.03, 147.53, 145.31, 136.56, 130.14, 120.87, 110.36, 108.16, 107.27, 101.23, 56.72, 52.54, 30.78, 27.62, 13.84. HRMS: m/z calculated for C_19_H_18_BrO_6_ (M-H^+^): 421.0283; found: 421.0231. IR υ max : 2359, 2341, 1715, 1577, 1489, 1240 cm^−1^.

#### Synthesis of methyl 2-(benzo[*d*][1,3]dioxol-5-yl)-7-methoxy-3-methyl-2,3-dihydrobenzofuran-5-carboxylate 16

In a 10-mililiter flask, 6 mg (0.014 mmol, 1 equiv.) of **15**, 0.3 mg (0.001 mmol, 0.1 equiv.) of CuI and 7 mg (0.02 mmol, 1.5 equiv.) of Cs_2_CO_3_ were dissolved in 300 μL of dry DMF under inert atmosphere. The mixture was heated at 130 °C for 16 h. Next, the crude was diluted with DCM, filtered and the solvent removed by vacuum. 5 mg of **16** were obtained as a slightly brownish powder (99% yield). ^1^H NMR (300 MHz, CDCl_3_) δ 7.59–7.43 (m, 2H), 6.92–6.84 (m, 2H), 6.79 (d, *J* = 7.8 Hz, 1H), 5.96 (s, 2H), 5.19 (d, *J* = 7.0 Hz, 1H), 3.93 (s, 3H), 3.90 (s, 3H), 3.53–3.37 (m, 1H), 1.42 (d, *J* = 7.0 Hz, 2H). ^13^C NMR (75 MHz, CDCl_3_) δ 167.07, 151.71, 148.17, 147.99, 144.07, 133.77, 133.06, 123.83, 120.41, 118.44, 113.35, 108.31, 106.84, 101.34, 94.41, 56.22, 52.13, 45.46, 29.84, 18.17. IR υ max : 1708, 1444, 1326, 1181 cm^−1^.

#### Synthesis of (2-(benzo[*d*][1,3]dioxol-5-yl)-7-methoxy-3-methyl-2,3-dihydrobenzofuran-5-yl)methanol 17

In a vial, 16 mg (0.05 mmol, 1 equiv.) of **16** and 8 mg (0.20 mmol, 4 equiv.) of LiAlH_4_ were dissolved in 500 μL of dry THF at 0 °C under inert atmosphere. The mixture was stirred at room temperature overnight. Then, the crude was diluted with AcOEt and 5 mL of deionized water were added. Three extractions with AcOEt were carried out and the organic phase was washed with NaCl (sat), dried over MgSO_4_, filtered and the solvent was removed by vacuum. 14 mg of a yellow oil were isolated as **17** (99% yield). ^1^H NMR (401 MHz, CDCl_3_) δ 6.92 (d, *J* = 1.7 Hz, 1H), 6.87 (dd, *J* = 8.0, 1.7 Hz, 1H), 6.83 (d, *J* = 1.6 Hz, 1H), 6.81–6.72 (m, 2H), 5.11 (d, *J* = 6.8 Hz, 1H), 4.63 (s, 2H), 3.89 (s, 3H), 3.56–3.35 (m, 3H), 1.38 (d, *J* = 6.8 Hz, 3H). ^13^C NMR (75 MHz, CDCl_3_) δ 148.07, 147.78, 147.09, 144.40, 134.69, 134.40, 133.26, 120.33, 114.98, 111.05, 108.24, 106.90, 101.26, 101.17, 93.61, 65.83, 56.17, 45.96, 29.84, 18.11. IR υ max : 3358, 1606, 1489, 1442, 1246, 1036 cm^-1^.

#### Synthesis of 2-(benzo[*d*][1,3]dioxol-5-yl)-7-methoxy-3-methyl-2,3-dihydrobenzofuran-5-carbaldehyde 18

In a vial, 14 mg (0.044 mmol, 1 equiv.) of **17** and 26 mg (0.27 mmol, 6 equiv.) of activated MnO_2_ were mixed in 200 μL of dry DCM. The mixture was stirred at room temperature for 16 h. Next, the crude was filter over silica and the solvent removed. **18** was obtained as a colorless oil (9 mg, 66% yield). ^1^H NMR (401 MHz, CDCl_3_) δ 9.84 (s, 1H), 7.37 (s, 1H), 7.33 (s, 1H), 6.91–6.84 (m, 2H), 6.80 (d, *J* = 7.9 Hz, 1H), 5.97 (s, 2H), 5.24 (d, *J* = 6.8 Hz, 1H), 3.95 (s, 3H), 3.57 - 3.44 (m, 1H), 1.44 (d, *J* = 6.8 Hz, 3H). ^13^C NMR (75 MHz, CDCl_3_) δ 190.76, 133.46, 131.68, 120.46, 120.22, 111.98, 108.38, 106.81, 101.40, 94.77, 56.26, 53.56, 45.22, 29.85, 18.24. IR υ max: 2921. 2851, 1731, 1681, 1445, 1321, 1251, 1133 cm^-1^.

#### Synthesis of (*E*)-5-(7-methoxy-3-methyl-5-(prop-1-en-1-yl)-2,3-dihydrobenzofuran-2-yl)benzo[d][1,3]dioxole (±)-1

*Carbonyl-olefin metathesis:* aldehyde **18** (0.012 mmol) and 2-ethyl-2-phenyl-1,3-dioxolane **19**^28,54^ (0.036 mmol) were introduced. Afterwards, 0.3 mL of dichloroethane (DCE) and 0.012 mmol of BF_3_·OEt_2_ were added and the reaction was stirred overnight at 70 °C. Conversion was measured by GC analysis which showed an approximately 50% conversion to the desired product. *Takai olefination:* To a stirring suspension of anhydrous CrCl_2_ (0.226 mmol, 8 equiv.) in 250 µL of dry THF, 18 μL (0.226 mmol, 8 equiv.) of dry DMF were added and the reaction was stirred at room temperature for 30 min. After that, a solution of 2-(benzo[*d*][1,3]dioxol-5-yl)-7-methoxy-3-methyl-2,3-dihydrobenzofuran-5-carbaldehyde (0.0282 mmol, 1 equiv.) and 1,1-diiodoethane (0.056 mmol, 2 equiv.) in 100 μL of dry THF was added and the reaction was stirred at room temperature for 1 h. Then, the mixture was purified by TLC using hexane:AcOEt 9:1 as an eluent. 8.5 mg of **1** were isolated as a white powder (93% yield). ^1^H NMR (300 MHz, CDCl_3_) δ 7.02–6.85 (m, 2H), 6.85–6.65 (m, 3H), 6.42–6.30 (m, 1H), 6.19–6.02 (m, 1H), 5.17–5.02 (m, 1H), 3.53–3.30 (m, 1H), 1.92 (dd, *J* = 7.2, 1.9 Hz, 0.6H), 1.87 (dd, *J* = 6.6, 1.6 Hz, 2.4H), 1.41–1.33 (m, 3H). ^13^C NMR (75 MHz, CDCl_3_) δ 148.05, 147.75, 146.66, 144.27, 134.49, 133.24, 132.38, 131.07, 130.00, 125.20, 123.63, 120.35, 113.51, 109.46, 108.21, 106.95, 101.24, 93.56, 56.11, 53.56, 53.31, 45.93, 34.51, 29.85, 18.51, 18.06. IR υ max : 2961, 2916, 2847, 2359, 1600, 1489, 1331, 1206 cm^−1^.

### Supplementary information

Supplementary_Information

Description of Additional Supplementary Files

Supplementary Data 1

## Data Availability

The datasets generated during and/or analysed during the current study are included in this published article (and its supplementary information files) or available from the corresponding author on reasonable request. Datasets could be also deposited in public repositories of the UPV and CSIC. Source data are provided with this paper. Supplementary Data 1 contains NMR copies.
