# Supplementary material for: Soluble individual metal atoms and ultrasmall clusters catalyze key synthetic steps of a natural product synthesis
Source: Commun Chem. 2024 Apr 4;7:76. doi: 10.1038/s42004-024-01160-z (PMC10995175; doi:10.1038/s42004-024-01160-z)
Supplement: Supplementary file 1 — Supplementary_Information [file 42004_2024_1160_MOESM1_ESM.pdf]

Supplementary Information (SI) for the manuscript:

Soluble individual metal atoms and ultrasmall clusters catalyze key synthetic steps of a natural product synthesis

Silvia Rodríguez-Nuévalos, Miguel Espinosa and Antonio Leyva-Pérez*

Instituto de Tecnología Química (UPV-CSIC), Universitat Politècnica de València-Consejo Superior de Investigaciones Científicas, Avda. de los Naranjos s/n, 46022 Valencia, Spain

Table of Contents

Supplementary Methods S2

Additional procedures and characterization data for compounds in Supplementary Figures S2

Supplementary Tables S1-S6 S10

Supplementary Figures S1-20 S12

Supplementary Methods

Additional procedures and characterization data for compounds in Supplementary Figures.

Sonogashira coupling between **6** and trimethylsilylacetylene under classical Pd and Cu-catalyzed reaction conditions (Fig. S4): In a 10-mililiter flask, 149 mg (0.40 mmol, 1 equiv.) of **6**, 15 mg (0.081 mmol, 0.2 equiv.) of CuI, 180 µL (1.21 mmol, 3 equiv.) of NEt_3 and 14 mg of $Pd(PPh_3)_2Cl_2$ were dissolved in 1.8 mL of dry 1,4-dioxane under inert atmosphere. Then, 80 µL (0.53 mmol, 1.3 equiv.) of trimethylsilylacetylene were added and the mixture was stirred at 80 ºC for 16 h. After cooling the reaction, 15 mL of AcOEt were added and the organic phase washed twice with HCl 0.5 M, once with NaCl (sat), dried with anhydrous $MgSO_4$, filtered and the solvent removed by vacuum. 130 mg of **7** as a brownish powder were obtained (94% yield).

Failed attempt to couple **8** with piperonyl alcohol **11** and give **S1** under Cu clusters-catalyzed reaction conditions (Fig. S5): In a 10-mililiter flask, 20 mg (0.07 mmol, 1 equiv.) of **8** were dissolved along with 17 mg (0.11 mmol, 1.5 equiv.) of piperonyl alcohol, 1.4 mg (0.007 mmol, 0.1 equiv.) of CuI and 48 mg (0.15 mmol, 2 equiv.) of Cs_2CO_3, in 500 µL of dry DMF under inert atmosphere, and heated at 120 °C for 16 h. The crude was diluted with AcOEt and some deionized water was added. Three extractions with AcOEt were carried out and the organic phase was washed with NaCl(sat), dried over $MgSO_4$, filtered and the solvent was removed by vacuum. The alcohol was fully recovered meanwhile the alkyne probably polymerize as it was also obtained a solid insoluble in $CDCl_3$ and DMSO-*d6*.

Failed attempt to couple **8** with piperonaldehyde and give **S3**. Some amounts of the trans-esterification product **S4** and piperonyl alcohol **11** were recovered after reaction (Fig. S6): In a 25-mililiter flask, 5 mL of a dissolution 1 M of Et_2AlCl (5 mmol, 1

equiv.) were heated at 60 °C along with 202 mg (5 mmol, 1 equiv.; 60 wt%) of NaH for 16 h under inert atmosphere. The reaction was cooled down to room temperature, filtered with a syringe in a vial and keep in the fridge under inert atmosphere. In a 25-mililiter flask, 3.7 mg (0.007 mmol, 0.03 equiv.) of Ni(dppp)Cl_2 and 180 μL of Et_2AlH (0.22 mmol, 1 equiv.) were dissolved in 220 μL of dry THF under inert atmosphere. The dissolution was cooled down to 0 °C and 58.7 mg (0.22 mmol, 1 equiv.) of **9** were added. The mixture was stirred for 3 h at room temperature. Then, 900 additional μL of dry THF and 65 μL (0.22 mmol, 1 equiv.) of Ti(OiPr)$_4$ were added. The reaction was cooled down to 0 °C and 1.1 mg of (0.004 mmol, 0.017 equiv.) *R*-BINOL and 11.2 mg (0.074 mmol, 0.33 equiv.) of piperonal were added. The reaction was stirred at room temperature overnight. Then, NH_4Cl was added to quench the reaction. Some HCl 2 M was also added and AcOEt. The organic phase was washed twice with HCl 2 M, once with NaCl (sat), dried over $MgSO_4$, filtered and the solvent removed by vacuum. After purifying the crude by TLC, using hexane:AcOEt 98:2 as an eluent, the transesterification of **7** with an isopropyl group (**S4**) was isolated along with the alcohol from piperonal (**11**). ^{1}H NMR of **S4** (300 MHz, $CDCl_3$) δ 7.79 (s, 1H), 7.51 (s, 1H), 5.32 - 5.16 (m, 1H), 3.96 - 3.94 (m, 3H), 1.38 (s, 6H).

Failed attempt to couple **9** with piperonaldehyde and give **S3** (Fig. S8): In a vial, 22.8 mg (0.15 mmol, 1.5 equiv.) of piperonal and 38 mg (0.10 mmol, 1 equiv.) of **7** were dissolved in dry DMF and cooled to -46 °C under inert atmosphere. Then, 14 μL (0.02 mmol, 0.2 equiv.) of ^{t}BuP4-base. The reaction was gently warmed up to room temperature for 5 h and stirred for 3 days. Next, the reaction was quenched by adding NH_4Cl (sat) and diluted with AcOEt. The organic phase was washed thrice with NH_4Cl (sat), twice with NaCl (sat), dried over $MgSO_4$, filtered and the solvent removed by vacuum. It was not any reaction, the starting materials were recovered.

Failed attempts to couple **9** with piperonaldehyde and give **S3**. Attempted reagents: a) Molecular sieves, THF; b) ^{t}BuOK, CuCl, THF; c) *n*BuLi, THF; d) TFA, Et_2O; e) $AlCl_3$, DCM; f) $Pd(OAc)_2$ (cat.), LiCl, DMF [a) and b) gave some **S5** product and c) gave some **S6** product] (Fig. S9): a) In a ten-mililiter flask, 25 mg (0.045 mmol, 1 equiv.) of **10** and 20 mg (0.135 mmol, 3 equiv.) of piperonal were dissolved in 500 μL of dry THF at room temperature under inert atmosphere. Some molecular sieves (4 Å were introduced in the flask). Then, 50 mg (0.05 mmol, 1.1 equiv.) of TBAF were added and the reaction was stirred at room temperature overnight. Next, the mixture was filtered and NH_4Cl and DCM were added. Three extractions with DCM were carried out. The organic phase was washed with NaCl (sat), dried over $MgSO_4$, filtered and the solvent was removed by vacuum. The crude was purify by TLC using hexane:AcOEt as an eluent. The aldehyde was recovered meanwhile **10** decompose to by-product **S5**. ^{1}H NMR of compound **S5** (401 MHz, $CDCl_3$) δ 7.40 (p, J = 2.0 Hz, 1H), 5.81 (d, J = 2.7 Hz, 0H), 5.54 (d, J = 2.6 Hz, 0H), 3.97 (s, 1H).

b) In a vial, 1.4 mg (0.009 mmol, 0.2 equiv.) of CuCl and 25 mg (0.045 mmol, 1 equiv.) of **10** were dissolved in 300 μL of dry THF under inert atmosphere. The reaction was cooled down to -15 °C and 60 μL (0.049 mmol, 1.1 equiv.; 1 M) of ^{t}BuOK were added. The mixture was stirred for 3.5 h and 12.8 mg (0.085 mmol, 1.7 equiv.) of piperonal dissolved in 100 μL of dry THF. The stirring was kept overnight at room temperature. Next, the reaction was diluted with AcOEt and washed twice with NH_4Cl (sat) and once with NaCl (sat). The organic phase was dried over $MgSO_4$, filtered and the solvent was removed by vacuum. The crude was purify by TLC using hexane:AcOEt 8:2 as an eluent. Compound **S5** was isolated and the aldehyde recovered.

c) In a 10-mililiter, 41 mg (0.07 mmol, 1 equiv.) of **10** were dissolved in 500 μL of dry THF and the dissolution was cooled down to -78 °C under inert atmosphere. Then, 32

μL (0.08 mmol, 1.1 equiv., 2.5 M) of *n*BuLi were added. After stirring for 1 h, 12 mg (0.08 mmol, 2.3 equiv.) of piperonal dissolved in 100 μL of dry THF along with 24 μL (0.08 mmol, 1.1 equiv.) of $Ti(OiPr)_4$ were added, previously mixed for 1 h. The temperature was gently rose to 0 °C for 40 min and, after that, the mixture was stirred overnight at room temperature. Next, the reaction was quenched with NH_4Cl (sat) and diluted with AcOEt. The organic phase was washed twice with NH_4Cl (sat), once with NaCl(sat), dried over $MgSO_4$, filtered and the solvent was removed by vacuum. The aldehyde was recovered meanwhile **10** underwent a partial dehalogenation from the aromatic ring, replacing the $SnBu_3$ group in the double bond, and giving rise to compound **S6** and some unidentified by-products. ^{1}H NMR of compound **S6** (401 MHz, $CDCl_3$) δ 6.81 (dd, J = 2.5, 1.5 Hz, 1H), 6.70 (t, J = 1.6 Hz, 1H), 6.61 (dd, J = 2.4, 1.5 Hz, 1H), 6.04 (d, J = 2.5 Hz, 1H), 5.40 (d, J = 2.5 Hz, 1H), 3.81 (s, 3H).

e) In a ten-mililiter flask, 25 mg (0.045 mmol, 1 equiv.) of **10** and 6.7 mg (0.045 mmol, 1 equiv.) of piperonal were dissolved in 400 μL of dry DCM at room temperature under inert atmosphere. Then, 6 mg (0.045 mmol, 1 equiv.) of $AlCl_3$ dissolved in 100 μL of dry DCM and added dropwise. The mixture was stirred for 16 h at room temperature. Next, the reaction was quenched with NH_4Cl (sat) and extracted three times with DCM. The organic phase was washed with NaCl (sat), dried over $MgSO_4$, filtered and the solvent was removed by vacuum. The crude was purify by TLC using hexane:AcOEt as an eluent. The desired product was not obtained.

f) In a vial, 25 mg (0.045 mmol, 1 equiv.) of **10**, 6.7 mg (0.045 mmol, 1 equiv.) of piperonal, 5.72 mg (0.14 mmol, 3 equiv.) and 0.5 mg (0.002 mmol, 0.05 equiv.) of $Pd(OAc)_2$ were dissolved in 200 μL of dry DMF at room temperature under inert atmosphere. This reaction was carried out twice, one in the absence of a base and one in the presence of DIPEA (24 μL, 0.14 mmol, 3 equiv.). The reactions were mixed 1 day

at room temperature and 2 days at 80 °C. Next, the mixtures were diluted with AcOEt, washed thrice with deionized water, dried over $MgSO_4$, filtered and the solvent was removed to vacuum. Any reaction was not observed.

Synthesis of benzoic acid **12** by Pinnick oxidation of piperonaldehyde (Fig. S10): In a 25-mililiter flask, 298.3 mg (2 mmol, 1 equiv.) of piperonal were dissolved along with 138.3 mg (0.8 mmol, 0.4 equiv., 70 wt%) of NaH_2PO_4 and 2 mL of H_2O_2 (21.2 mmol, 10.6 equiv., 30 wt%) in 12 mL of a mixture MeCN:H_2O 5:1. The temperature was lowered to 0 ºC and 368 mg (4 mmol, 2 equiv.) of $NaClO_2$ dissolved in deionized water were added dropwise. The mixture was stirring at room temperature overnight. 20 mL of HCl 2 M were added and extracted three times with AcOEt. Next, three extractions with NaOH 2 M were carried out and acidified with HCl(c), until pH 2, approximately. Finally, three additional extractions with AcOEt were done. This organic phase was washed twice with NaCl (sat), dried over anhydrous $MgSO_4$, filtered and the solvent removed by vacuum. 220 mg of **12** were isolated as a slightly brown powder (68% yield).

Synthesis of vinyl iodide **S7** from **10** and failed attempt to couple **S7** with piperonaldehyde and give **S3** after a Nozaki-Hiyama-Kishi coupling. Some amounts of the de-stannate product **S8** were recovered after reaction (Fig. S11): In a 10-mililiter flask, 100 mg (0.18 mmol, 1 equiv.) of **10** and 68 mg (0.27 mmol, 1.5 equiv.) of I_2 were dissolved in 2.6 mL of dry DCM under inert atmosphere, overnight. Next, the crude was purify by column chromatography using hexane:AcOEt 97.5:2.5 as an eluent. 62 mg of **S7** were obtained as a yellow oil (87%yield). ^{1}H NMR (300 MHz, $CDCl_3$) δ 7.59 (d, J = 1.9 Hz, 1H), 7.46 (d, J = 1.9 Hz, 1H), 6.37 - 5.99 (m, 2H), 3.97 (s, 3H), 3.93 (s, 3H). Then, to a dissolution of 87 mg (0.70 mmol, 4 equiv.) of $CrCl_2$ in 600 µL of dry DMF, 70 mg (0.18 mmol, 1 equiv.) of **S7** and 32 mg (0.21, 1.2 equiv.) of piperonal dissolved

in 700 μL of dry DMF were added under inert atmosphere. After mixing overnight, the reaction was quenched with deionized water and extracted three times with DCM. The organic phase was washed with NaCl (sat), dried over $MgSO_4$, filtered and the solvent was removed by vacuum. The desired reaction did not take place, the only product found was **S8**. ^1H NMR of compound **S8** (300 MHz, $CDCl_3$) δ 7.85 (d, *J* = 1.8 Hz, 2H), 7.45 (dd, *J* = 5.0, 1.8 Hz, 1H), 7.11 (dd, *J* = 17.4, 11.0 Hz, 1H), 5.80 (dd, *J* = 17.4, 1.0 Hz, 1H), 5.44 (dd, *J* = 11.0, 1.0 Hz, 1H), 3.96 (s, 3H), 3.94 (s, 3H).

Failed attempt to couple **10** with acyl chloride **13** and give **14** with $Pd_{2\text{-}3}$ clusters as a catalyst (Fig. S12): In a vial, 16 mg (0.03 mmol, 1 equiv.) of **10** were dissolved along with 0.38 mg ($3.3\cdot10^{-4}$ mmol, 0.01 equiv.) of $Pd(PPh_3)_4$ and 5.6 mg (0.03 mmol, 1 equiv.) of **13** in 100 μL of DMF dry, also containing 11.7 mg (0.033 mmol, 1.1 equiv.) of Cs_2CO_3. The reaction was heated at 120 °C for 16 h. Then, the reaction was quenched with deionized water and extracted thrice with AcOEt. The organic phase was washed with NaCl (sat), dried over $MgSO_4$, filtered and the solvent removed by vacuum.

Failed attempt of intermolecular carbonyl-olefin metathesis reaction between aldehyde **18** and ketal **S12** (Fig. S14): To a solution with 6.25 mmol of 2-bromomethyl-1,3-dioxolane **S10** in 25 mL of dry diethyl ether at -78 ºC, 6.5 mmol of *n*-butyllithium were added dropwise and the reaction was left stirring to rt. Afterwards, 6.25 mmol of methyl iodide were added along with a 21 mg of iodine and reaction was stirred until completion. Then, the resulting 2-ethyl-1,3-dioxolane **S12** was distilled form the reaction mixture and used in the next step without further purification. In a 1 mL vial, aldehyde **18** (0.012 mmol) and **S12** (0.036 mmol) were introduced. Afterwards, 0.3 mL of DCE and 0.012 mmol of $BF_3\cdot OEt_2$ were added, and the reaction was stirred overnight at 70 ºC.

Validation of the structure of **1** after a successful Takai reaction between **18** and ethyldiiodide **S15**, the latter prepared from a halogen exchange reaction between ethyldibromide **S13** and *n*-butyl iodide **S14** catalyzed by zeolite NaX (Fig. S15, synthesis of ethyl diiodide **S15**, for the Takai coupling procedure see the main text): In a 25-mililiter round-bottomed flask, 483 μL (5.3 mmol, 1 equiv.) of dibromoethane **S13** were heated at 130 °C along with 6 mL (53 mmol, 10 equiv.) of BuI **S14** and 10 mg of zeolite Na-Y (CBV-100) for 19 h. Then, the mixture was filtrated and the excess of **S14** evaporated. The crude was analyzed by GC and GC-MS, obtaining a 1:4 **S14**:**S15** mixture. This crude was directly used in next reaction.

Supplementary Tables

Table S1 EXAFS results for Pd(0) foil and Pd_1 individual atoms in solution.

Sample	NPd-Pd	σ^2 (Å^2)	ΔE0 (eV)	R
**Pd foil**	12 (fixed)	0.005 ±0.0004	3.03 ± 1.01	2.736 ± 0.007
**Pd in solution**	6.25 ± 1.62	0.006 ±0.002	3.56 ±1.80	2.759 ± 0.013

Table S2 Estimated size of the different clusters employed in this study, according to the Jellium model.

Sample	Range of wavelength (nm)	N
**Cu**	285 – 329	4 – 7
	492 - 499	21 - 23
**Pd**	285 – 305	4 – 6
	425 - 519	14 - 26
**Pt**	285 – 320	2 – 5
	335 - 420	13 -27

Table S3 Estimated Cu clusters structural formulae from MALDI-TOF analysis.

Formula	m/z experimental	m/z calculated
**$Cu_6(DMF)(H^+)$**	453.8542	453.6384
**$Cu_6(DMF)_3$**	598.7983	598.7342
**Cu_4I_2**	504.9556	504.4821
**$Cu_7(O)(CO_3^{-2})(H_2O)$**	554.506	554.9074
**$Cu_8(O)_4(DMF)$**	642.8873	642.4674
**$Cu_8(O)_3(DMF)(H_2O)$**	642.8873	642.4848
**$Cu_{12}(H_2O)(H^+)$**	774.7848	774.1731
**$Cu_{17}(CO_3^{-2})(H^+)$**	1190.8180	1190.7804

Table S4 Estimated Pd clusters structural formulae from MALDI-TOF analysis.

Formula	m/z experimental	m/z calculated
$Pd_3(NMP)(AcO^-)(H^+)$	476.9857	476.8958
$Pd_4(H_2O)_2(H^+)$	461.0117	461.7307
$Pd_6(H_2O)_2(H^+)$	672.0361	672.4490
$Pd_5(NMP)(AcO^-)(H^+)$	688.0103	688.7028
$Pd_6(NMP)_2(H_2O)$	877.0467	877.0045
$Pd_6(NMP)(AcO^-)_2(H_2O)_2(H^+)$	893.0190	893.8640
$Pd_6(NMP)_2(H_2O)_2(OH^-)$	893.0190	892.9990

Table S5 Estimated Pt clusters structural formulae from MALDI-TOF analysis.

Formula	m/z experimental	m/z calculated
$Pt_2(H2O)_2(O)$	442.1057	441.9443
$Pt_2(C_4H_9SiO)(H_2O)_2(H^+)$	532.0789	532.0703
$Pt_3(O)_3$	631.1475	631.9131
$Pt_3(C_4H_9SiO)(H_2O)_5$	775.0411	775.9872
$Pt_4(H_2O)_4$	874.1098	873.9801
$Pt_4[(C_4H_9Si)_2O]$	964.0834	964.8940
$Pt_5(H_2O)_4(OH^-)$	893.0190	892.9990

Table S6 Concentration of several metals (ppm) during the reduction of **14** to **15** (Sample A purified, Sample B non-purified), as measured by ICP-OES analyses.

Metal/Sample	A	B
**Sn**	0.143	7.474
**Pt**	0.043	0.075
**B**	81.575	90.806
**Si**	760.004	770.96
**Cu**	0.037	0.109
**Pd**	0	0.008
**Na**	93.564	93.012
**K**	6.876	6.58

Supplementary Figures

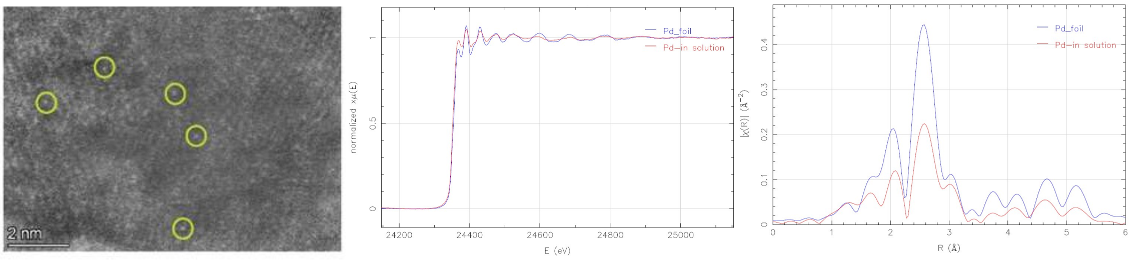

Fig. S1. Characterization of Pd_1 individual atoms in the benzyl alcohol solution. From left to right: A representative aberration-corrected high-angle annular dark field scanning-transmission electron microscopy (AC-HAADF-STEM) image of the Pd species in solution, after being trapped in active charcoal (some Pd individual atoms are marked with yellow circles); X-ray absorption near-edge structure (XANES) and extended X-ray absorption fine structure (EXAFS) spectra of the solution (red lines) compared to Pd foil (blue lines).

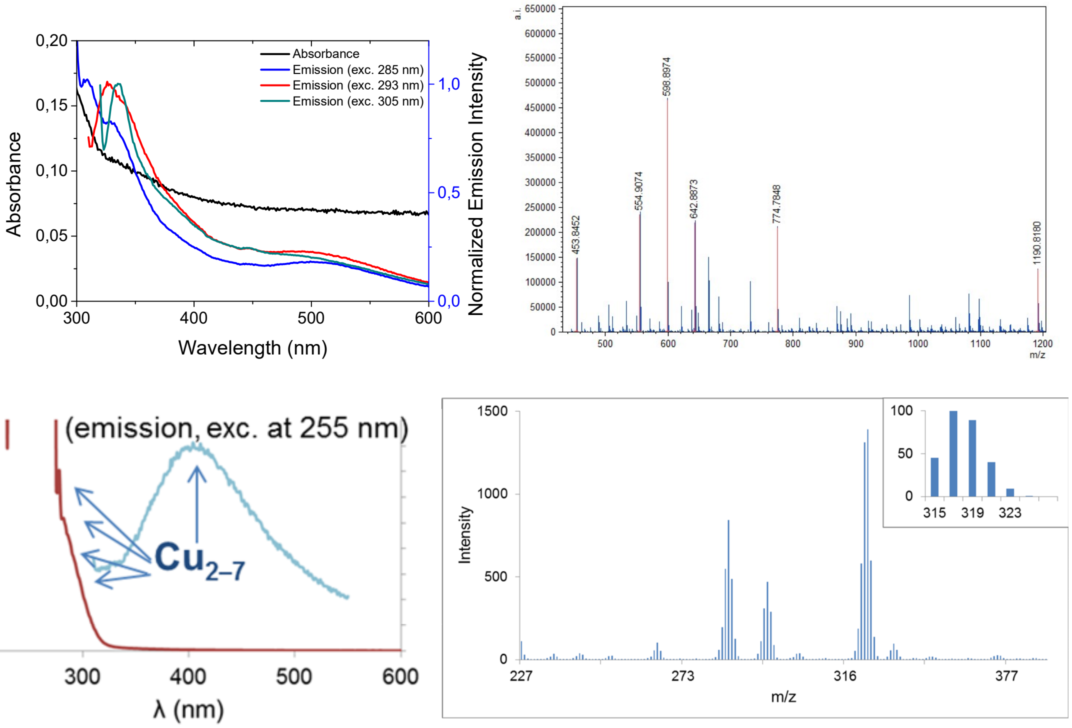

Fig. S2. Top: Characterization of the Cu clusters in DMF synthesized during this work, using CuI as the catalyst precursor. From left to right: The absorption and emission spectra of the metal species under reaction conditions, and the MALDI-TOF analysis of the clusters. Bottom: Characterization of $Cu_{2\text{-}7}$ clusters. From left to right: Absorption (red line) and emission (blue line) ultraviolet-visible (UV-vis) spectrophotometric

spectra of Cu clusters in DMF using CuI as the catalyst precursor, showing the expected wide fluorescence band for 2-7 atoms clusters according to the Jellium model; and ESI-TOF spectrum of small (Cu_5) clusters in ethanolic solution in negative ion mode, together with the simulation of the relative intensity peaks.

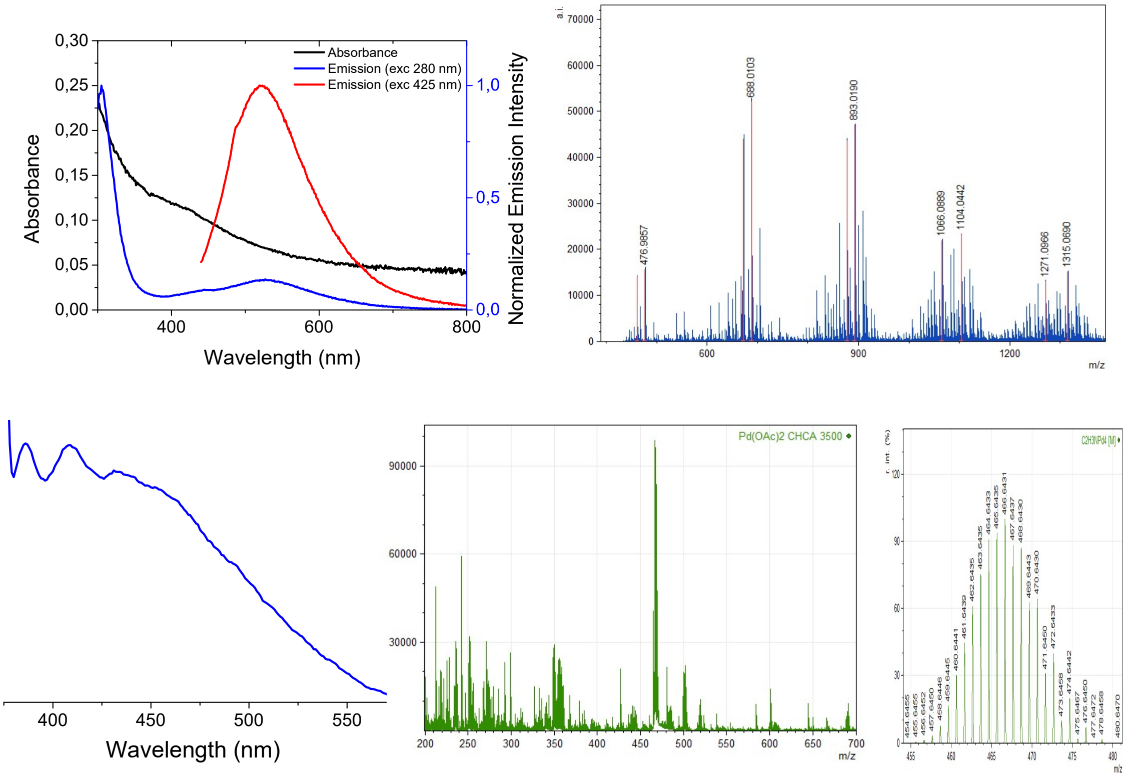

Fig. S3. Top: Characterization of Pd clusters in NMP synthesized during this work, using $Pd(AcO)_2$ as precursor. From left to right: The absorption and emission spectra of the metal species under reaction conditions, and the MALDI-TOF analysis of the clusters. Bottom: Characterization of Pd_{2-3} clusters. From left to right: Emission ultraviolet-visible (UV-vis) spectrophotometric measurement of $Pd(OAc)_2$ in aqueous NMP after irradiation at a wavelength of 370 nm, showing the expected fluorescence bands for this ultrasmall size of clusters; matrix-assisted laser desorption/ionization coupled to time-of-flight (MALDI-TOF) mass spectrometry spectra for $Pd(OAc)_2$ in acetonitrile solution and the corresponding simulation of the spectrum for a $Pd_4(CH_3CN)$ cluster

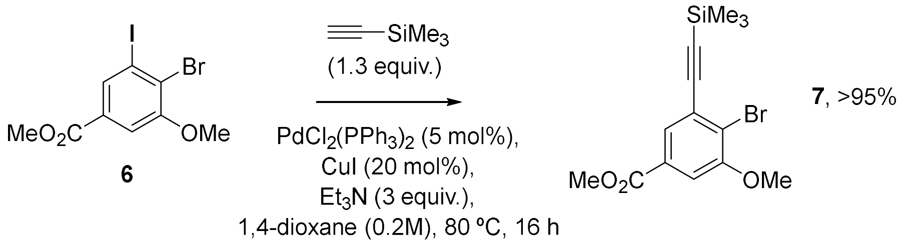

Fig. S4. Sonogashira coupling between **6** and trimethylsilylacetylene under classical Pd and Cu-catalyzed reaction conditions.

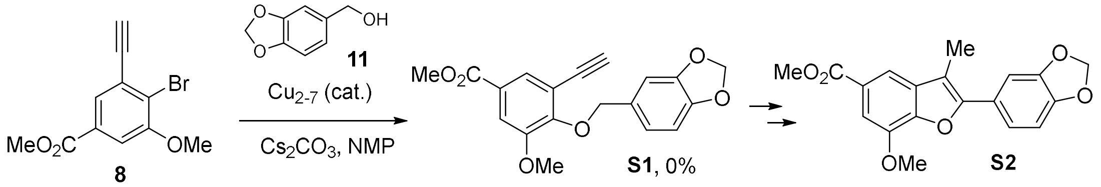

Fig. S5. Failed attempt to couple **8** with piperonyl alcohol **11** and give **S1** under Cu clusters-catalyzed reaction conditions.

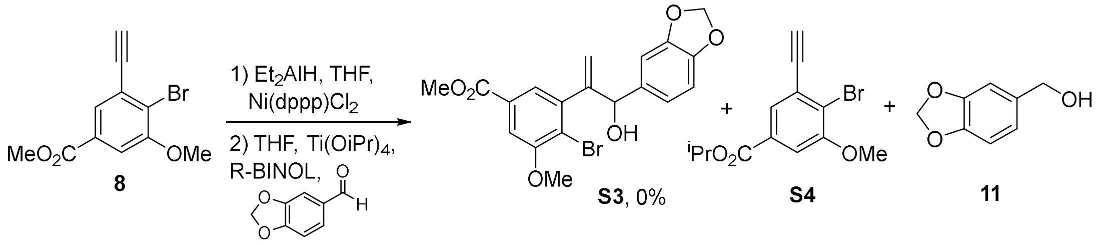

Fig. S6. Failed attempt to couple **8** with piperonaldehyde and give **S3**. Some amounts of the trans-esterification product **S4** and piperonyl alcohol **11** were recovered after reaction.

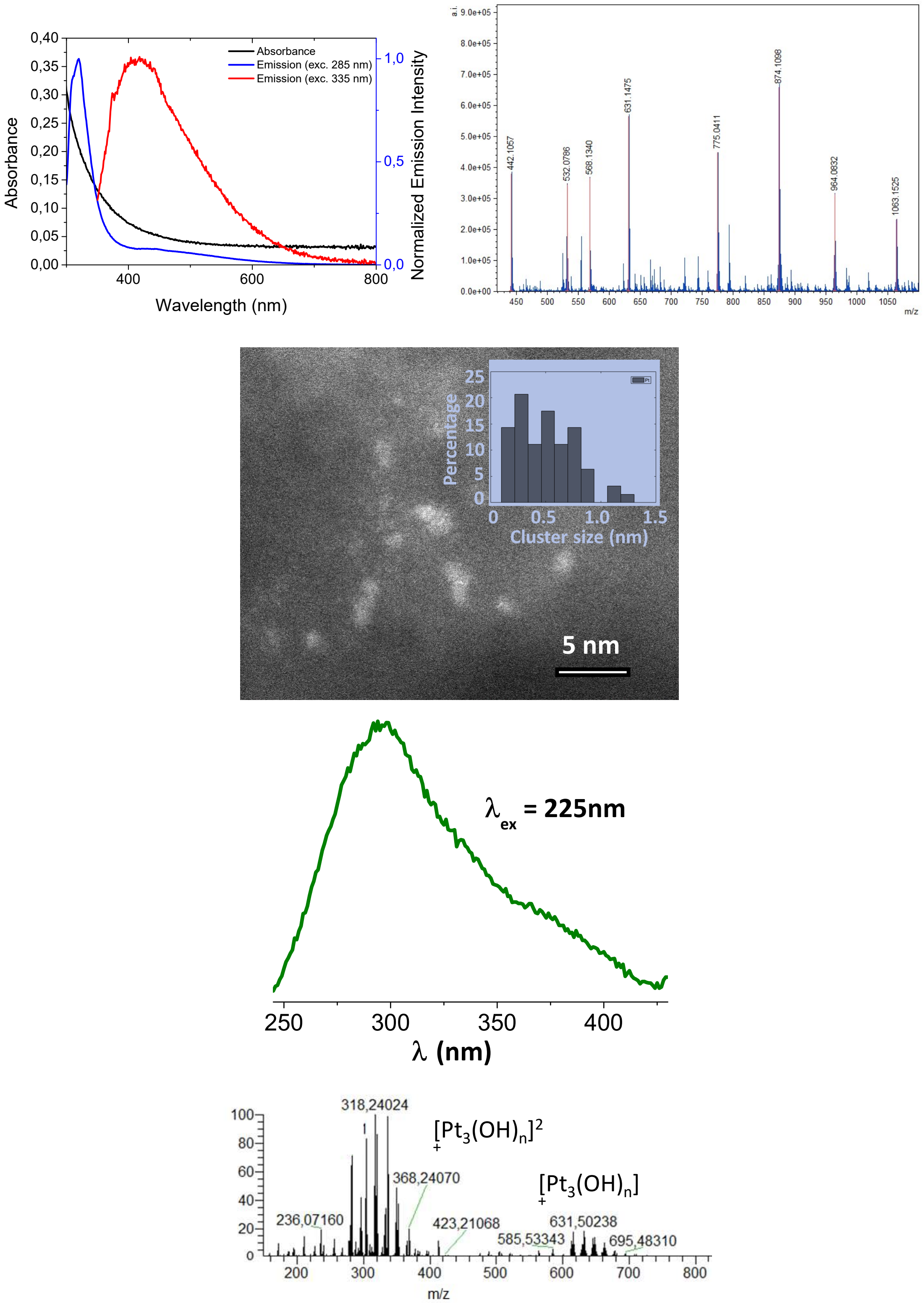

Fig. S7. Top: Characterization of Pt clusters in toluene synthesized during this work, using the Karstedt's catalyst as precursor. From left to right: The absorption and emission spectra of the metal species under reaction conditions, and the MALDI-TOF analysis of the clusters. Bottom: Characterization of $Pt_{3\text{-}5}$ clusters. From top to bottom:

A representative aberration-corrected high-angle annular dark field scanning-transmission electron microscopy (AC-HAADF-STEM) image of the Pt clusters in solution with the corresponding cluster size distribution histogram; emission spectrum after irradiating the solution with Pt clusters at 225 nm; high-resolution mass spectrum of Pt clusters in solution, obtained using an ORBITRAP instrument.

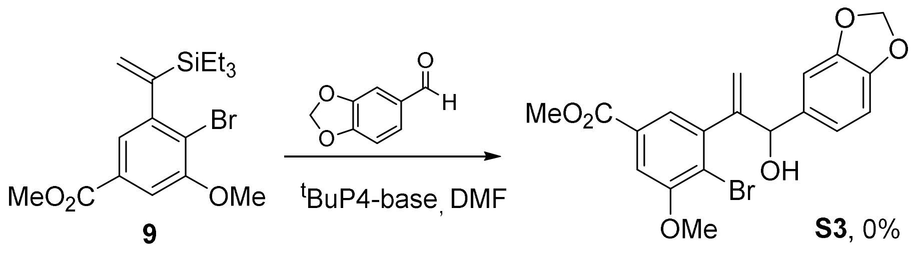

Fig. S8. Failed attempt to couple **9** with piperonaldehyde and give **S3**.

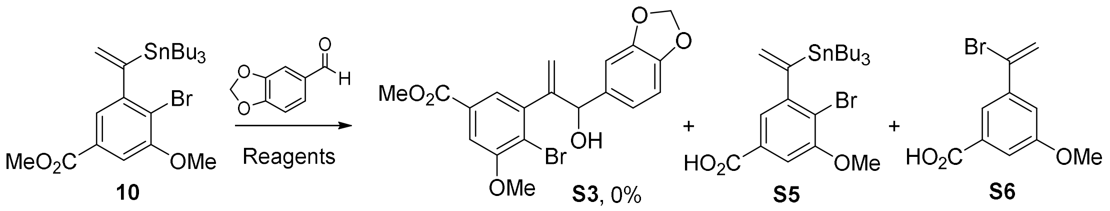

Fig. S9. Failed attempts to couple **9** with piperonaldehyde and give **S3**. Attempted reagents: a) Molecular sieves, THF; b) ^{t}BuOK, CuCl, THF; c) *n*BuLi, THF; d) TFA, Et_2O; e) $AlCl_3$, DCM; f) $Pd(OAc)_2$ (cat.), LiCl, DMF [a) and b) gave some **S5** product and c) gave some **S6** product].

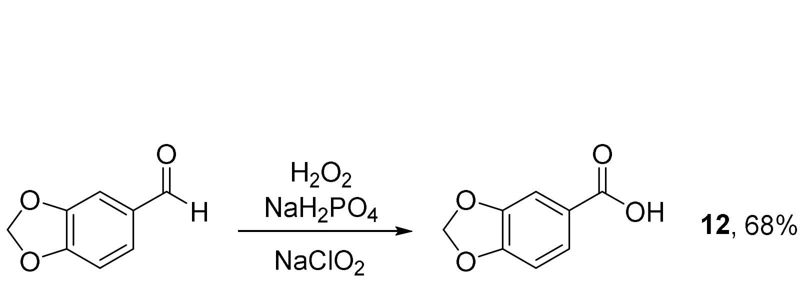

Fig. S10. Synthesis of benzoic acid **12** by Pinnick oxidation of piperonaldehyde.

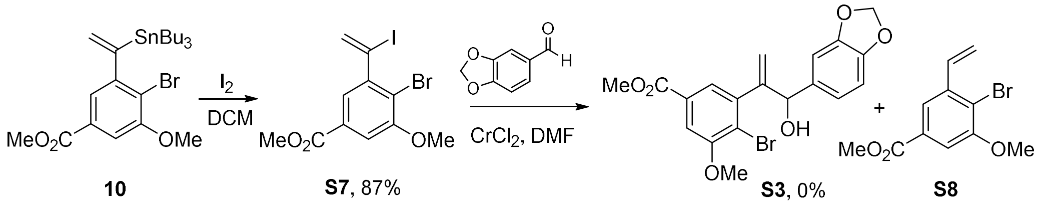

Fig. S11. Synthesis of vinyl iodide **S7** from **10** and failed attempt to couple **S7** with piperonaldehyde and give **S3** after a Nozaki-Hiyama-Kishi coupling. Some amounts of the de-stannate product **S8** were recovered after reaction.

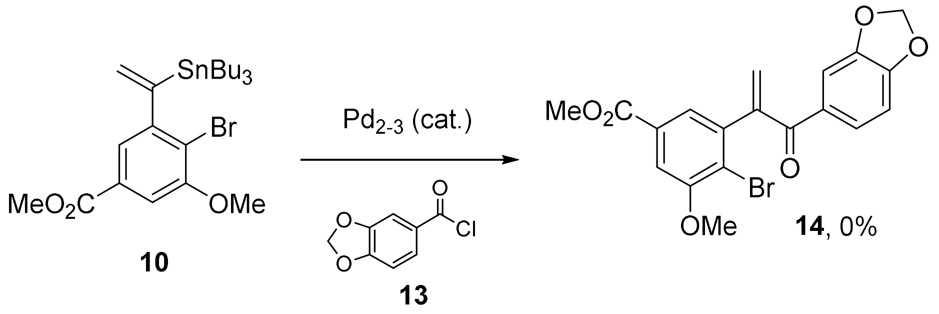

Fig. S12. Failed attempt to couple **10** with acyl chloride **13** and give **14** with $Pd_{2\text{-}3}$ clusters as a catalyst.

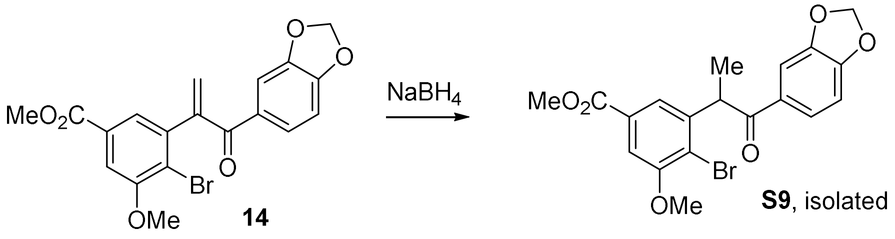

Fig. S13. Isolation of ketone **S9** after selective hydrogenation of the alkene in **14** with $NaBH_4$.

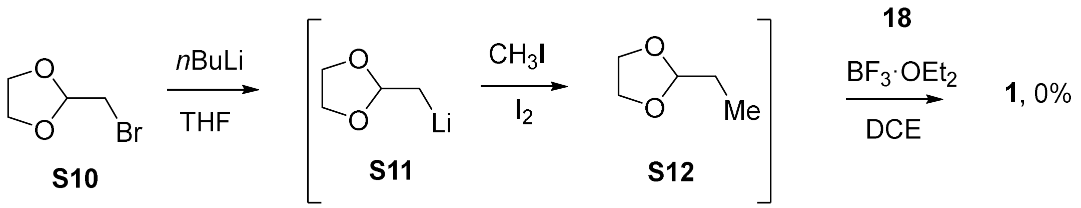

Fig. S14. Failed attempt of intermolecular carbonyl-olefin metathesis reaction between aldehyde **18** and ketal **S12**.

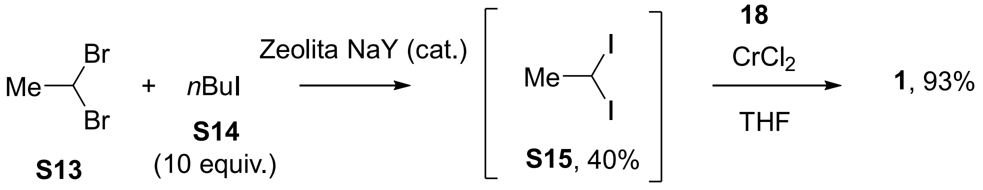

Fig. S15. Validation of the structure of **1** after a successful Takai reaction between **18** and ethyldiiodide **S15**, the latter prepared from a halogen exchange reaction between ethyldibromide **S13** and *n*-butyl iodide **S14** catalyzed by zeolite NaX.

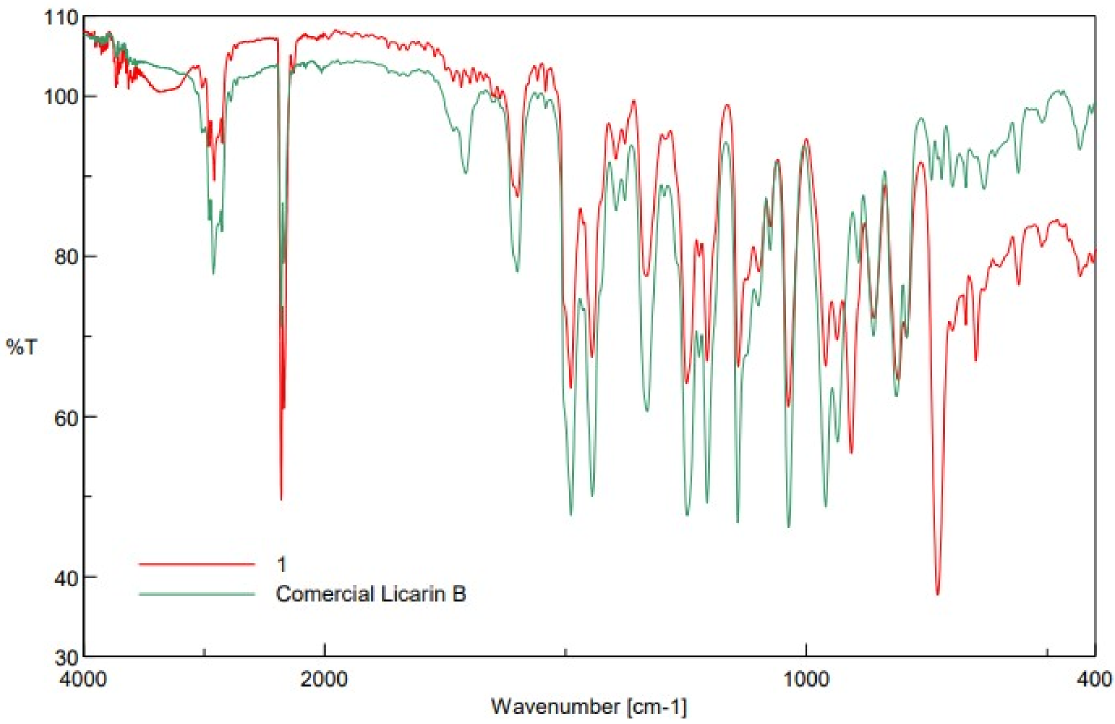

Fig. S16. Comparison of the Fourier transform infrared spectra (FT-IR) for the synthesized (±)-Licarin B **1** and a commercial sample.

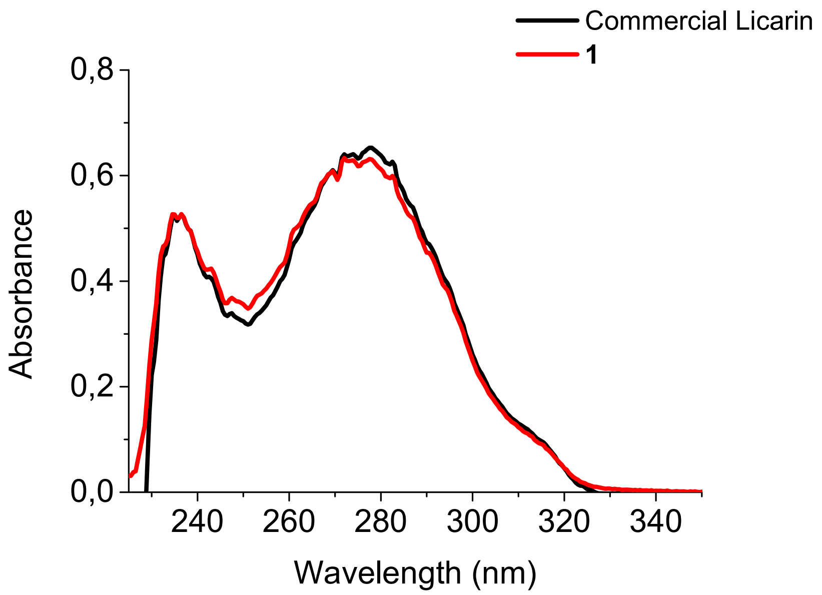

Fig. S17. Comparison of the absorption UV-vis spectrophotometric spectra for the synthesized (±)-Licarin B **1** and a commercial sample (10 µM in DCM).

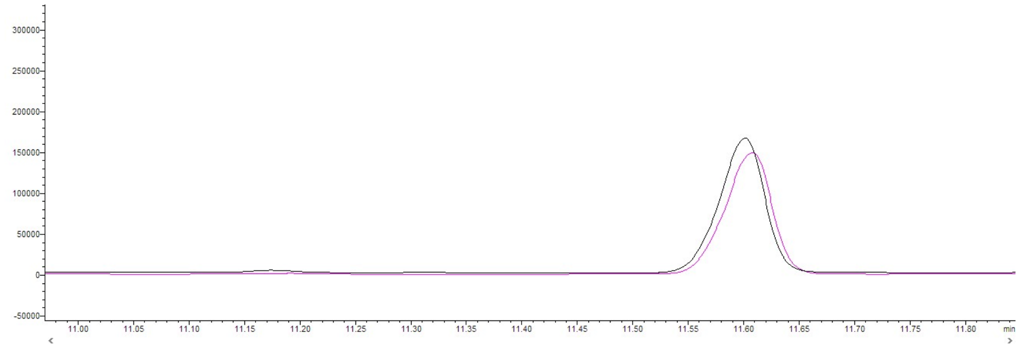

Fig. S18. Comparison of the gas chromatograms (GC) for the synthesized (±)-Licarin B **1** (black line) and a commercial sample (pink line).

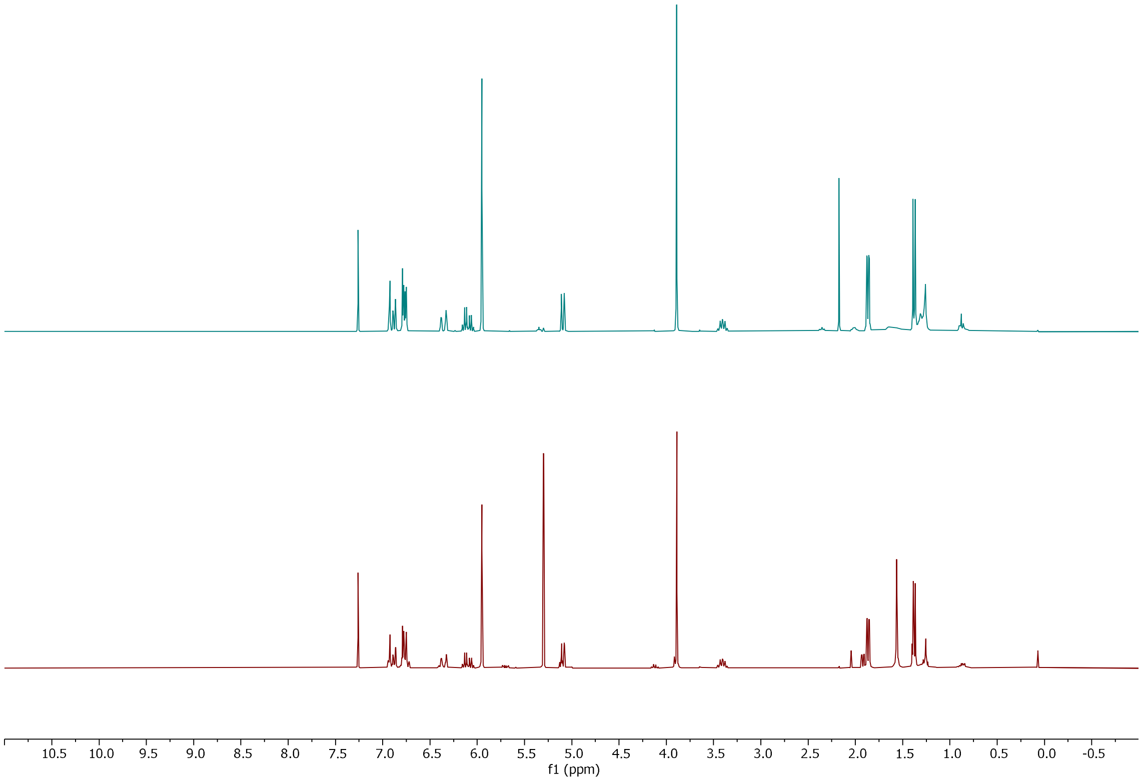

Fig. S19. Comparison of the ^{1}H nuclear magnetic resonance (NMR) spectra for the synthesized (±)-Licarin B **1** (red, bottom) and a commercial sample (blue, top).

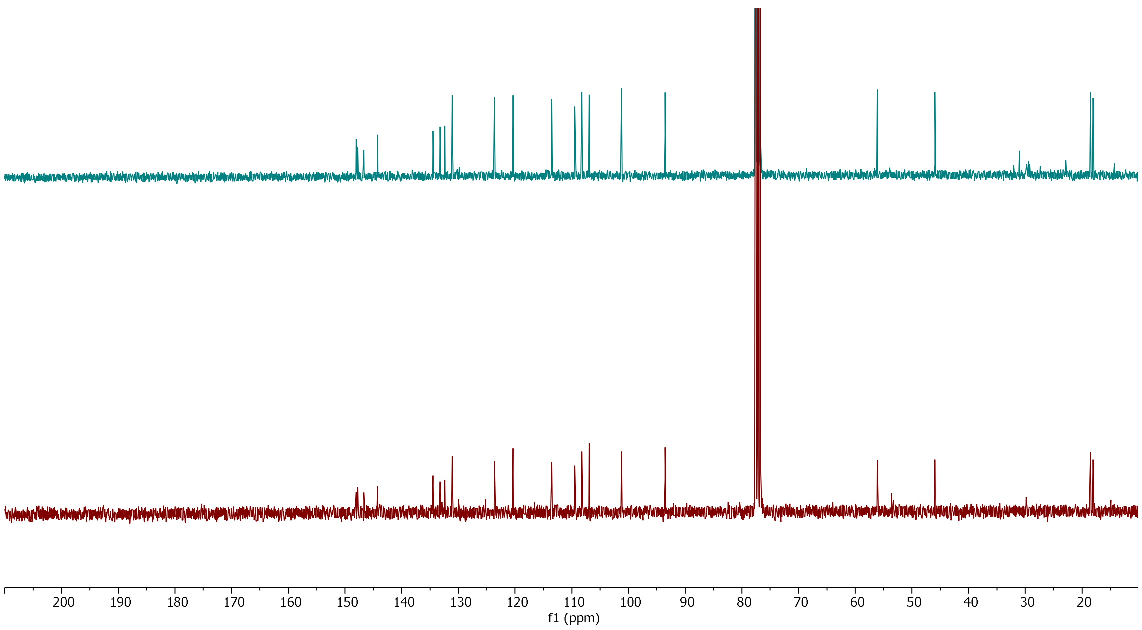

Fig. S20. Comparison of the ^{13}C nuclear magnetic resonance (NMR) for the synthesized (±)-Licarin B **1** (red, bottom) and a commercial sample (blue, top).
