# Supplementary material for: Soluble individual metal atoms and ultrasmall clusters catalyze key synthetic steps of a natural product synthesis
Source: Commun Chem. 2024 Apr 4;7:76. doi: 10.1038/s42004-024-01160-z (PMC10995175; doi:10.1038/s42004-024-01160-z)
Supplement: Supplementary file 3 — Supplementary Data 1 [file 42004_2024_1160_MOESM3_ESM.pdf]

**Supplementary Data (SD) for the manuscript:**

# Soluble individual metal atoms and ultrasmall clusters catalyze key synthetic steps of a natural product synthesis

Silvia Rodríguez-Nuévalos, Miguel Espinosa and Antonio Leyva-Pérez*

Instituto de Tecnología Química (UPV-CSIC), Universitat Politècnica de València-Consejo Superior de Investigaciones Científicas, Avda. de los Naranjos s/n, 46022 Valencia, Spain

## NMR Copies

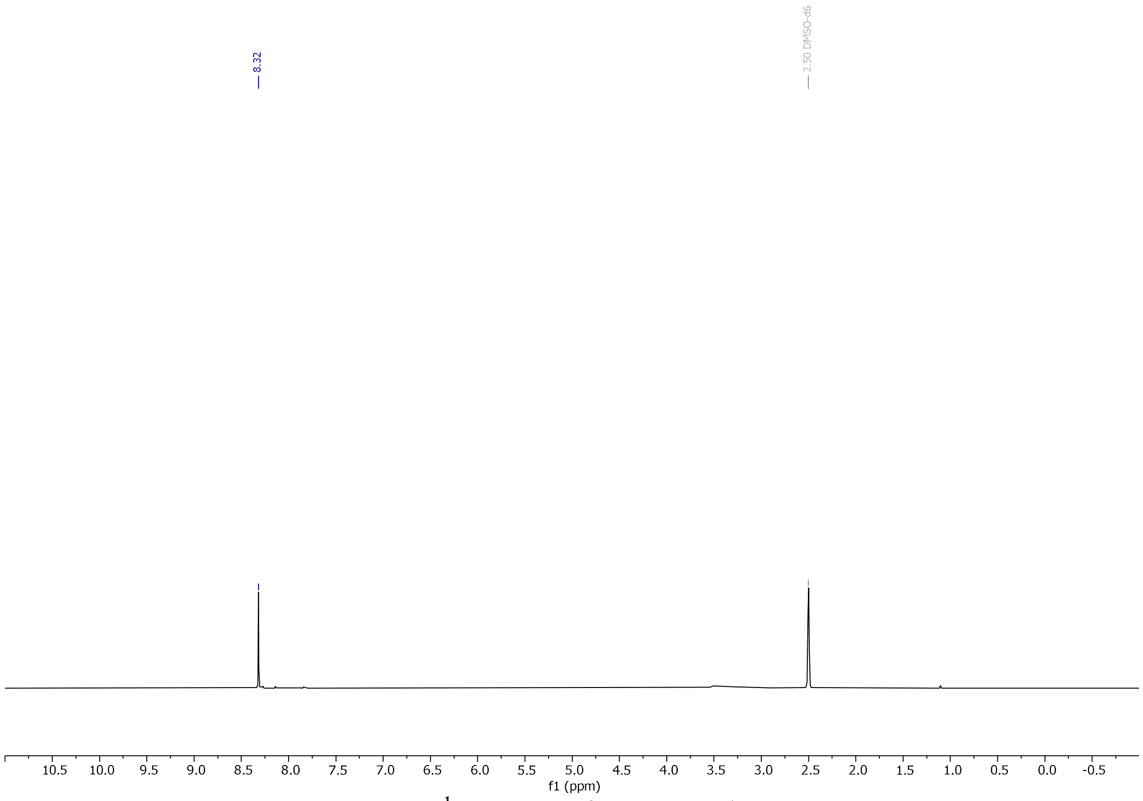


**Fig. S21**. $^{1}$H NMR of compound **4**

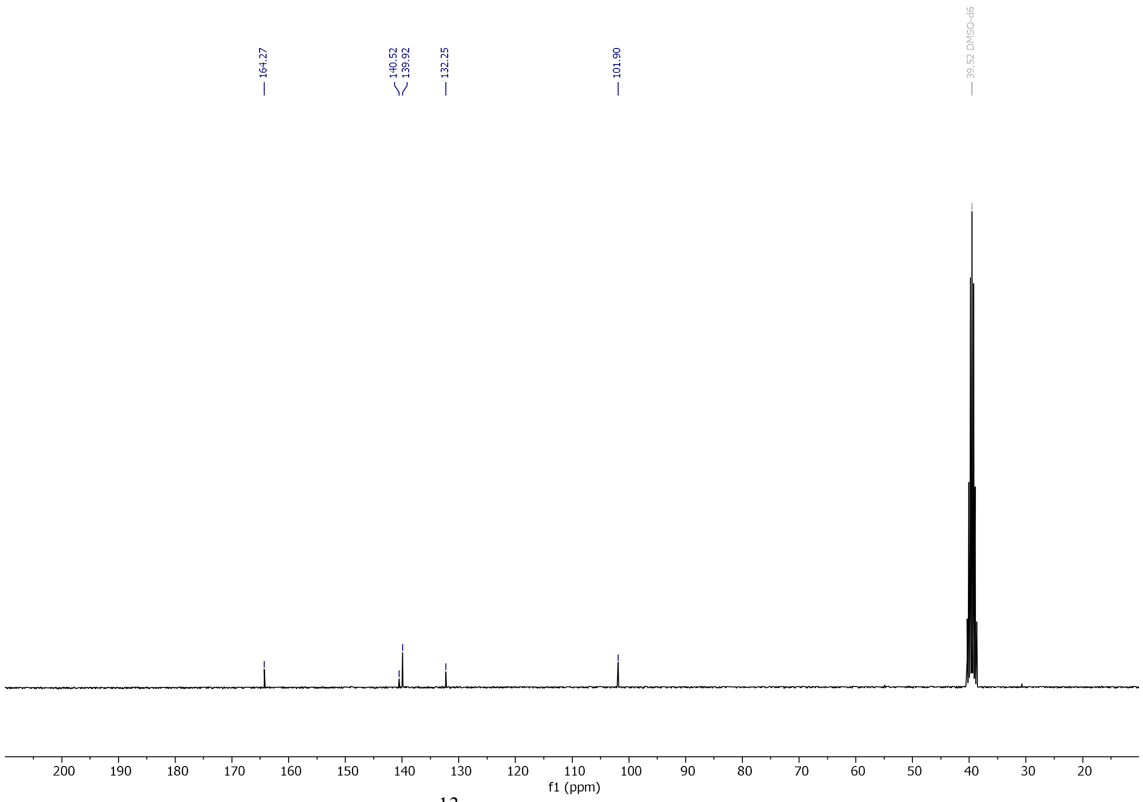


**Fig. S22**. $^{13}$C NMR of compound **4**

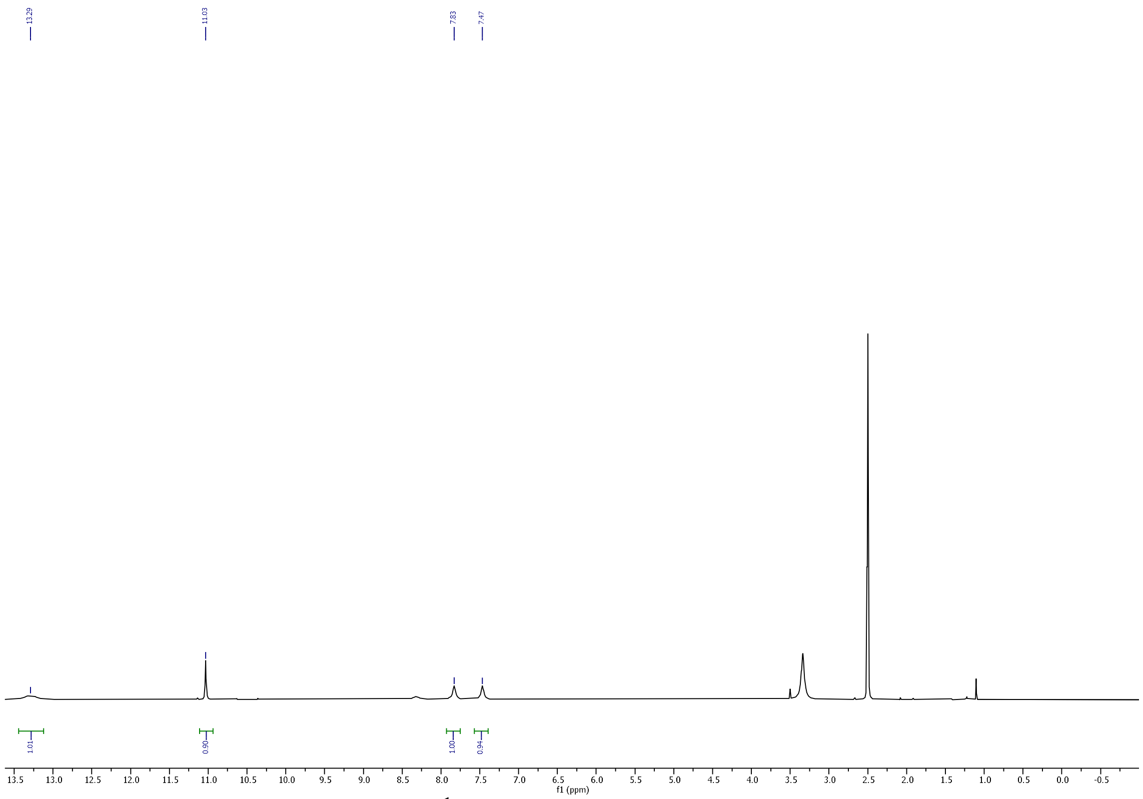


**Fig. S23**. $^{1}$H NMR of compound **5**

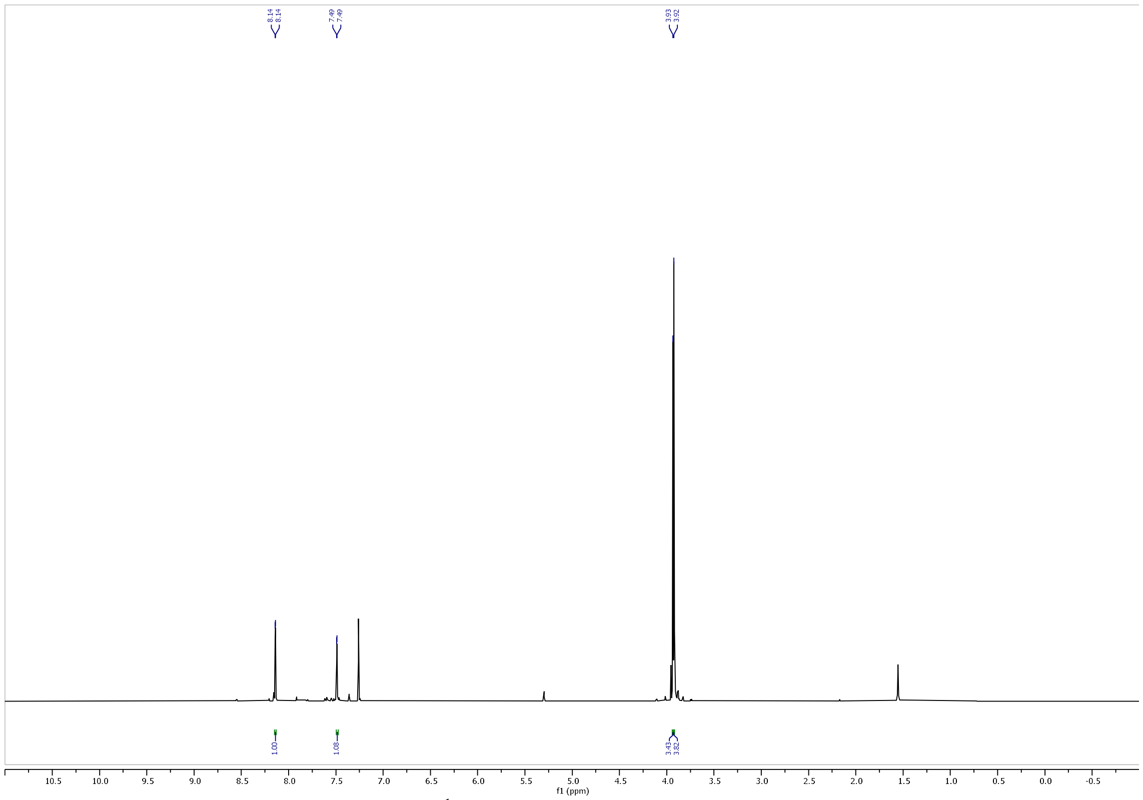

**Fig. S24**. $^{1}$H NMR of compound **6**

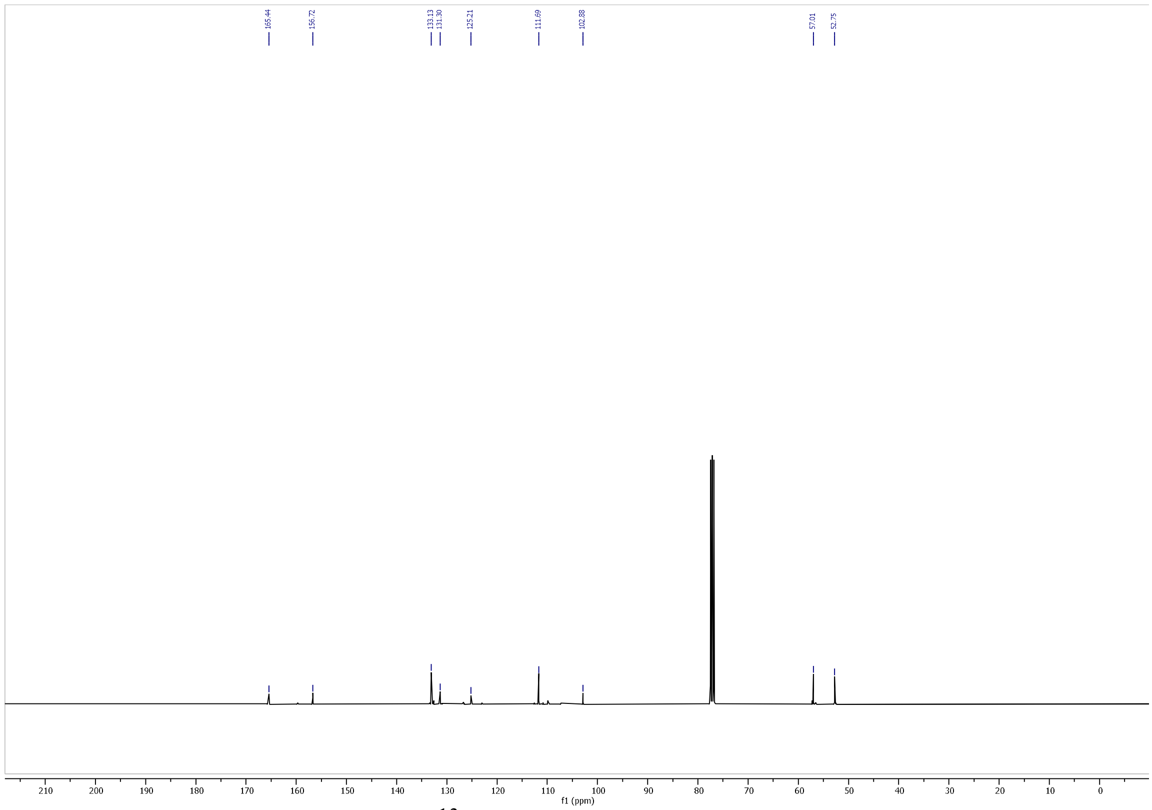

**Fig. S25**. $^{13}$C NMR of compound **6**

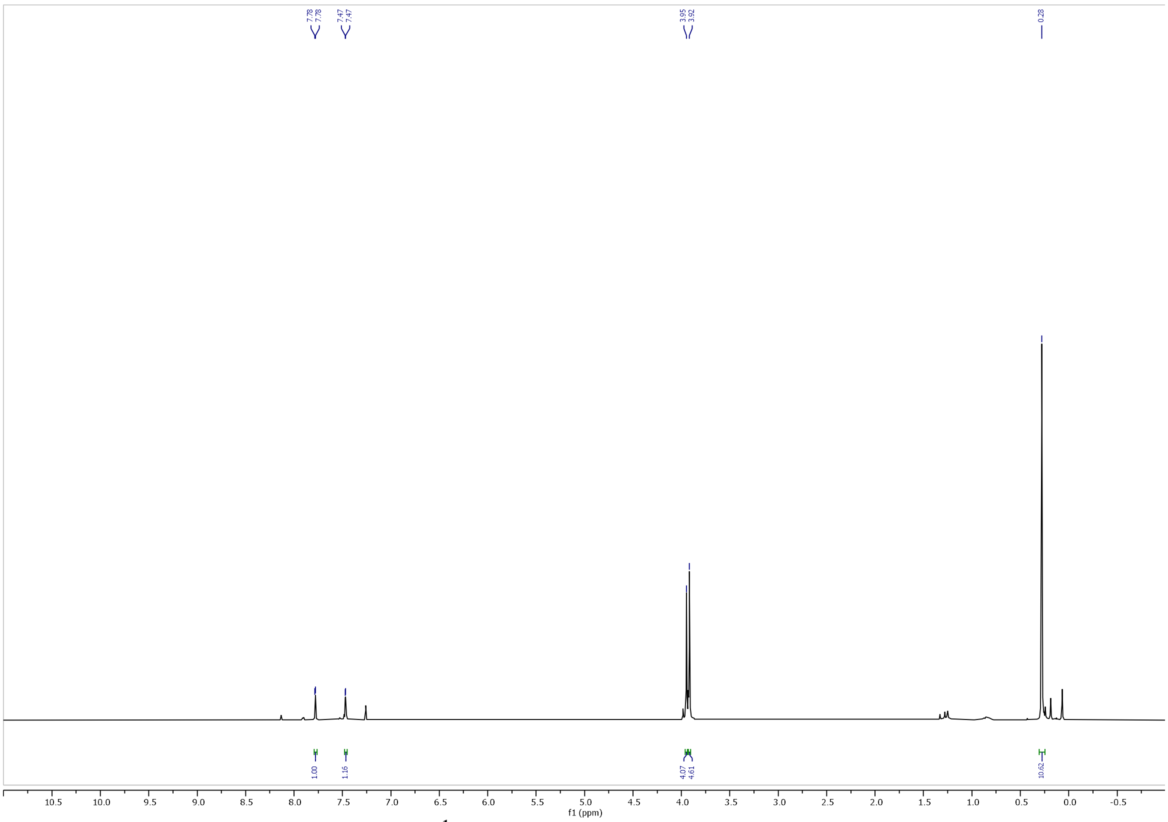

**Fig. S26**. $^{1}$H NMR of compound **7**

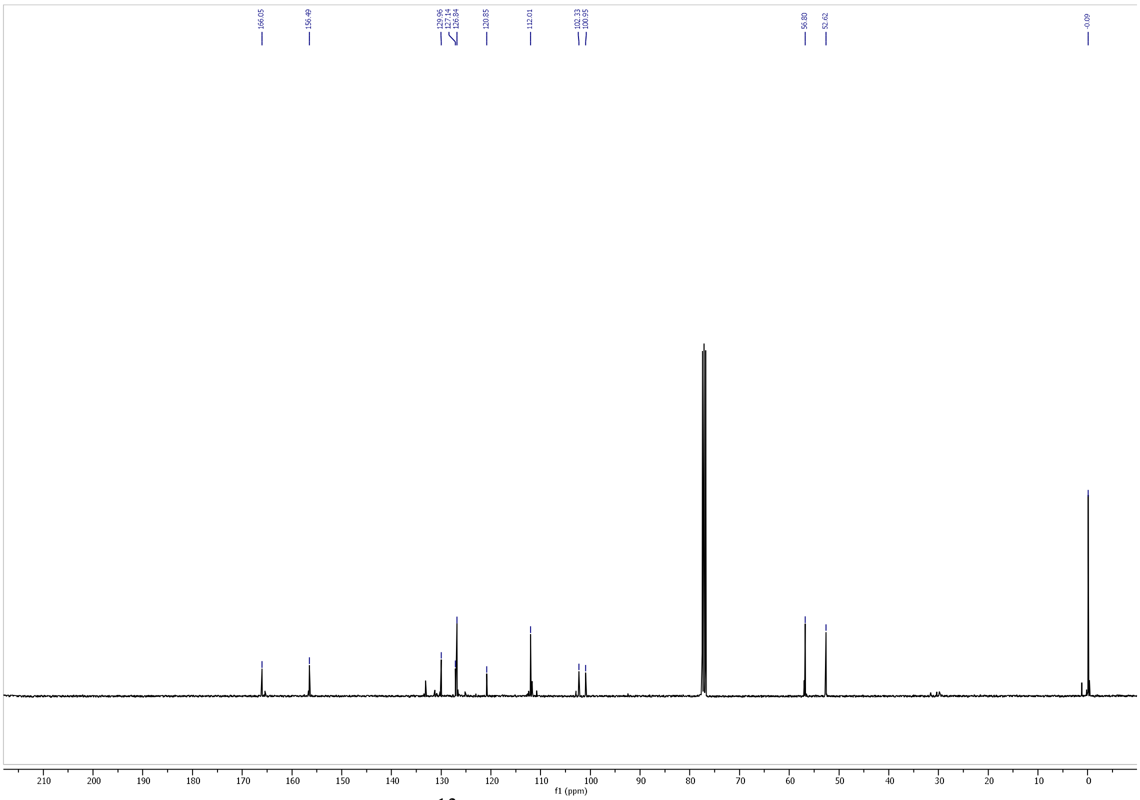

**Fig. S27**. $^{13}$C NMR of compound **7**

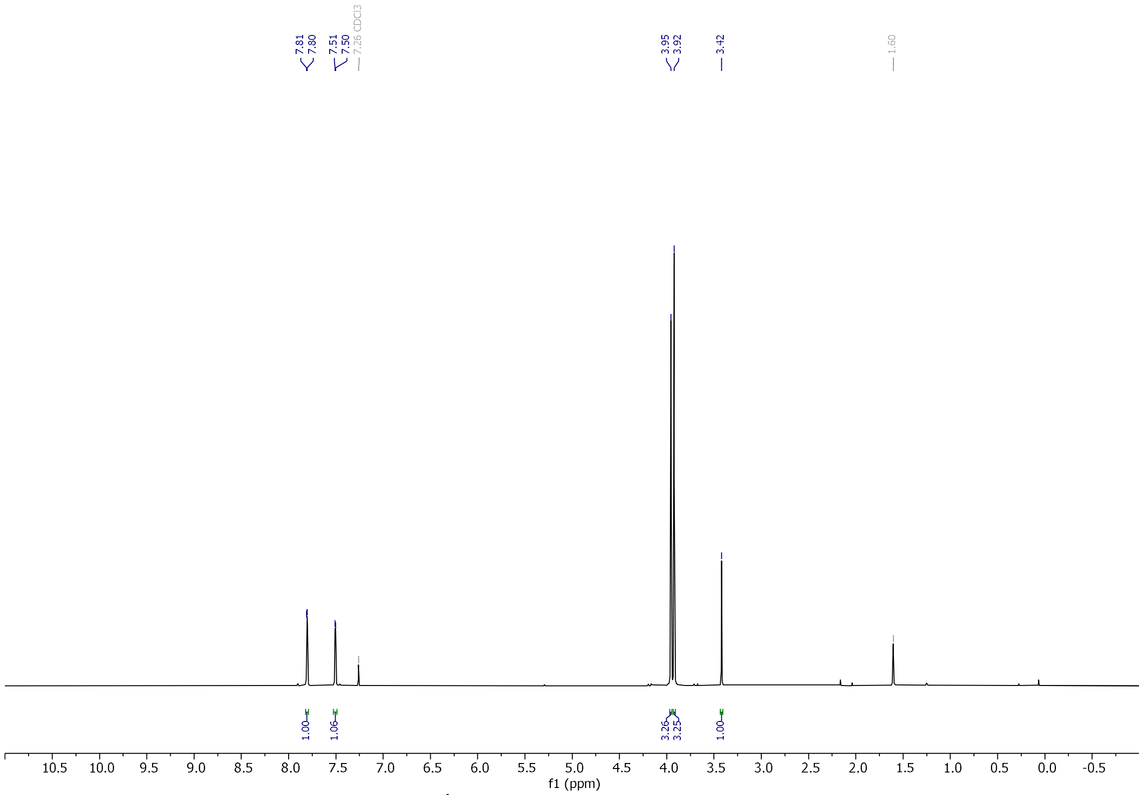


**Fig. S28**. $^{1}$H NMR of compound **8**

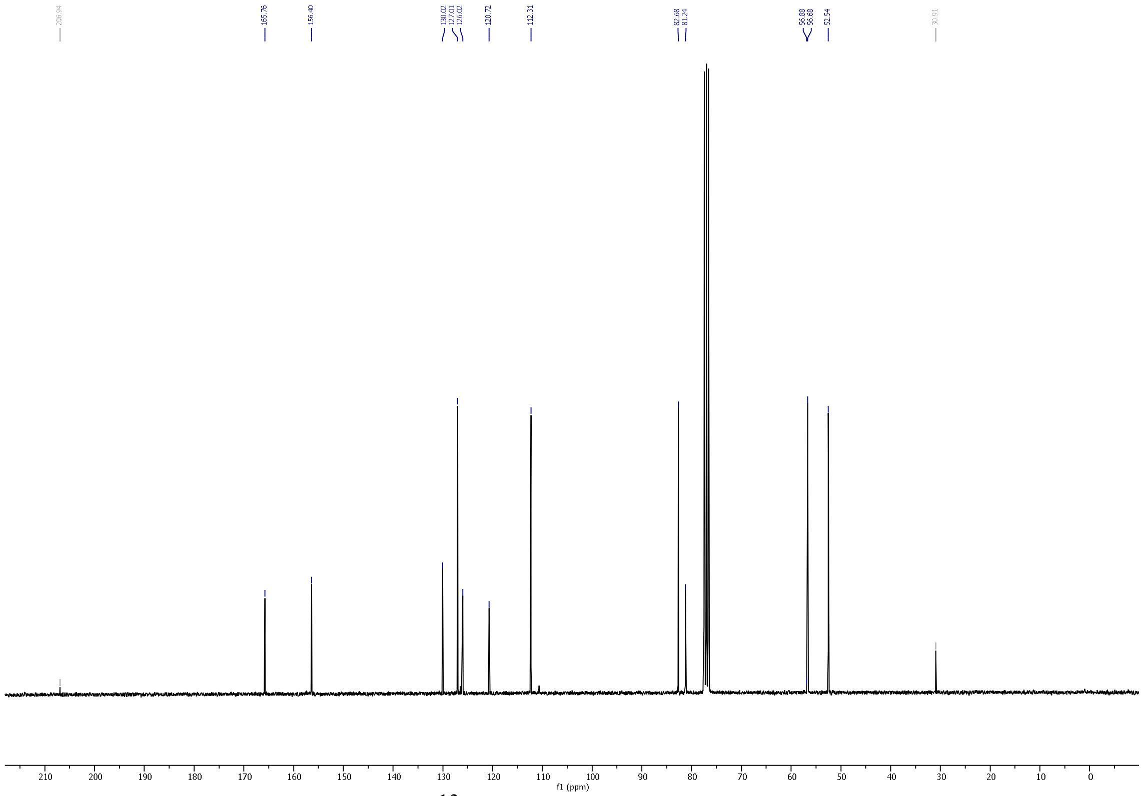


**Fig. S29**. $^{13}$C NMR of compound **8**

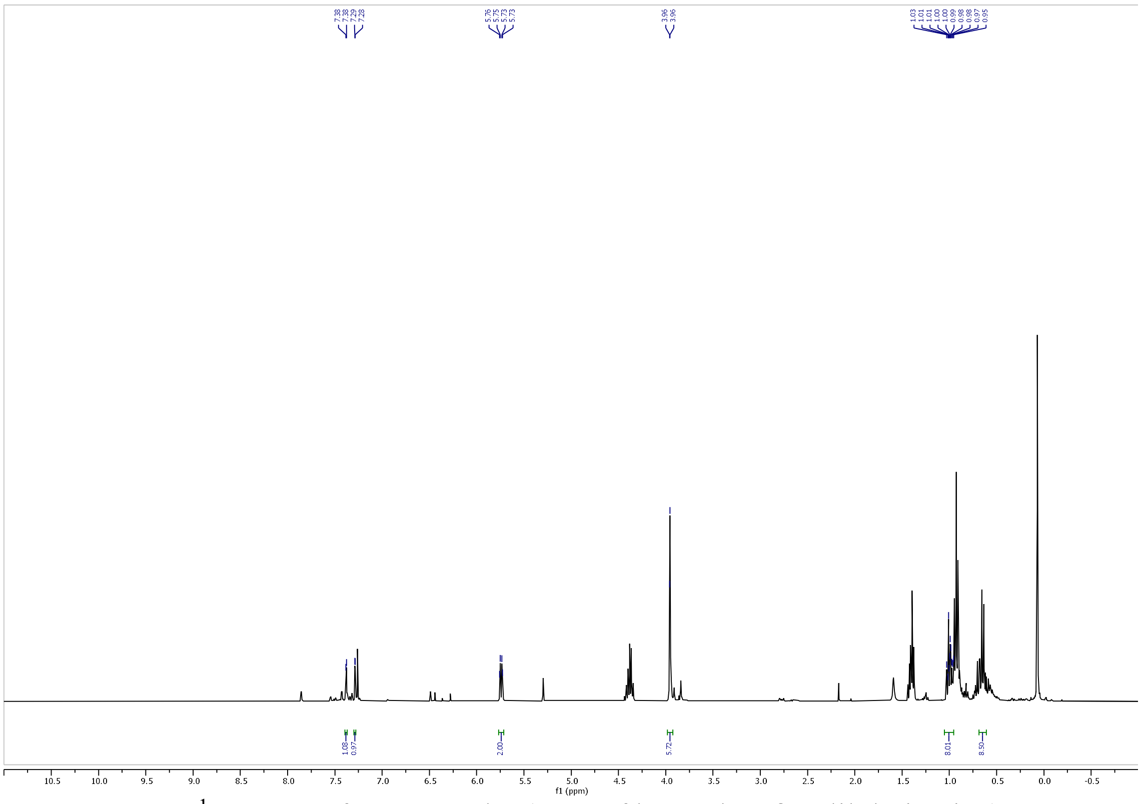


**Fig. S30**. $^{1}$H NMR of compound **9** (25% of byproduct β-sylil derivative)

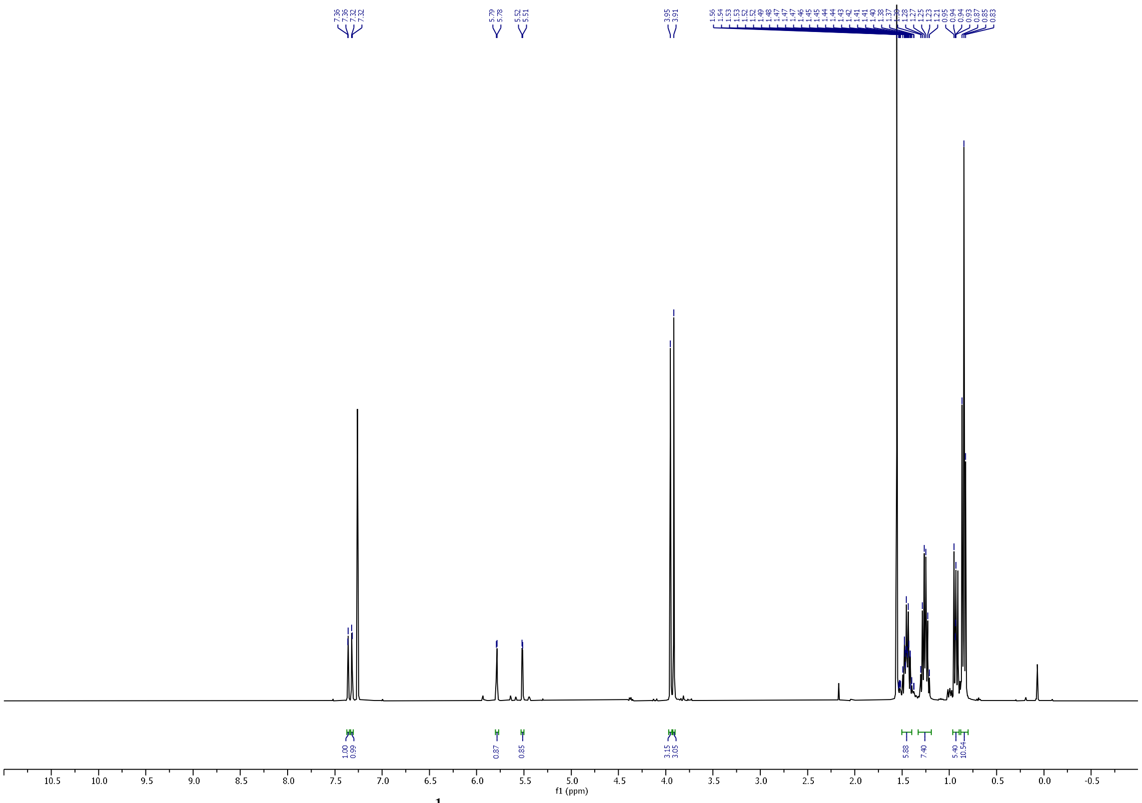


**Fig. S31**. $^{1}$H NMR of compound **10**

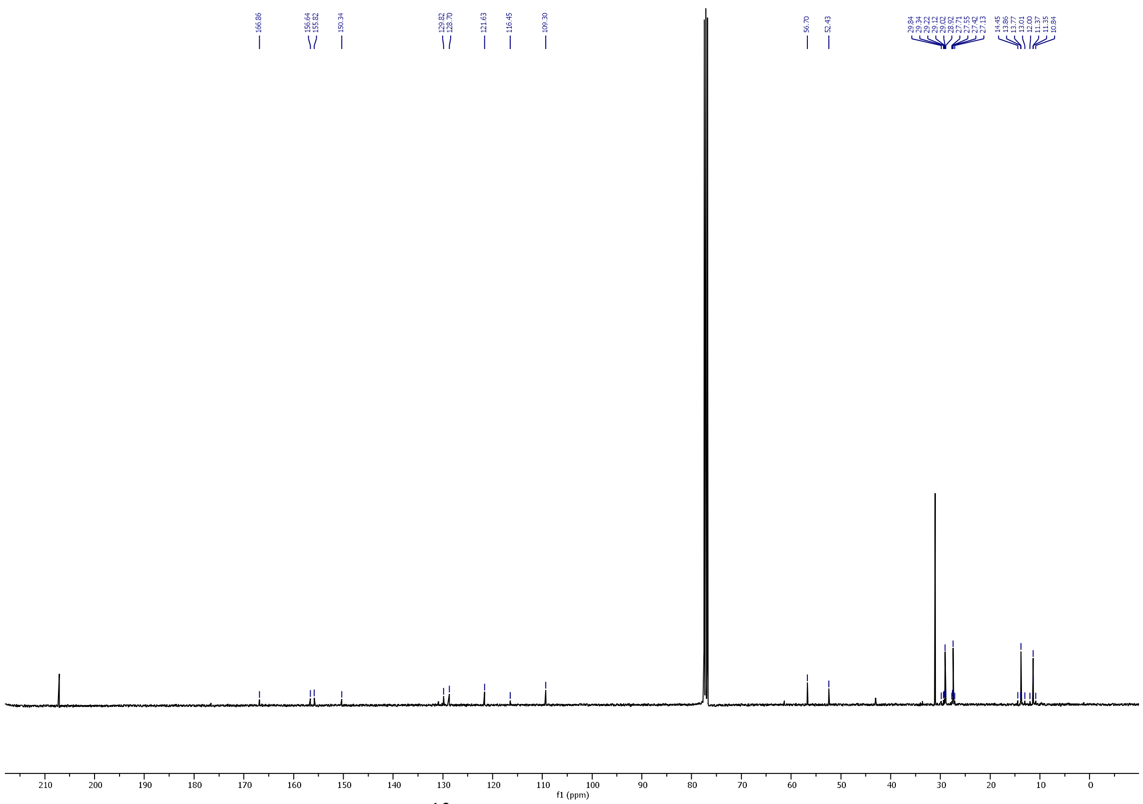


**Fig. S32**. $^{13}$C NMR of compound **10**

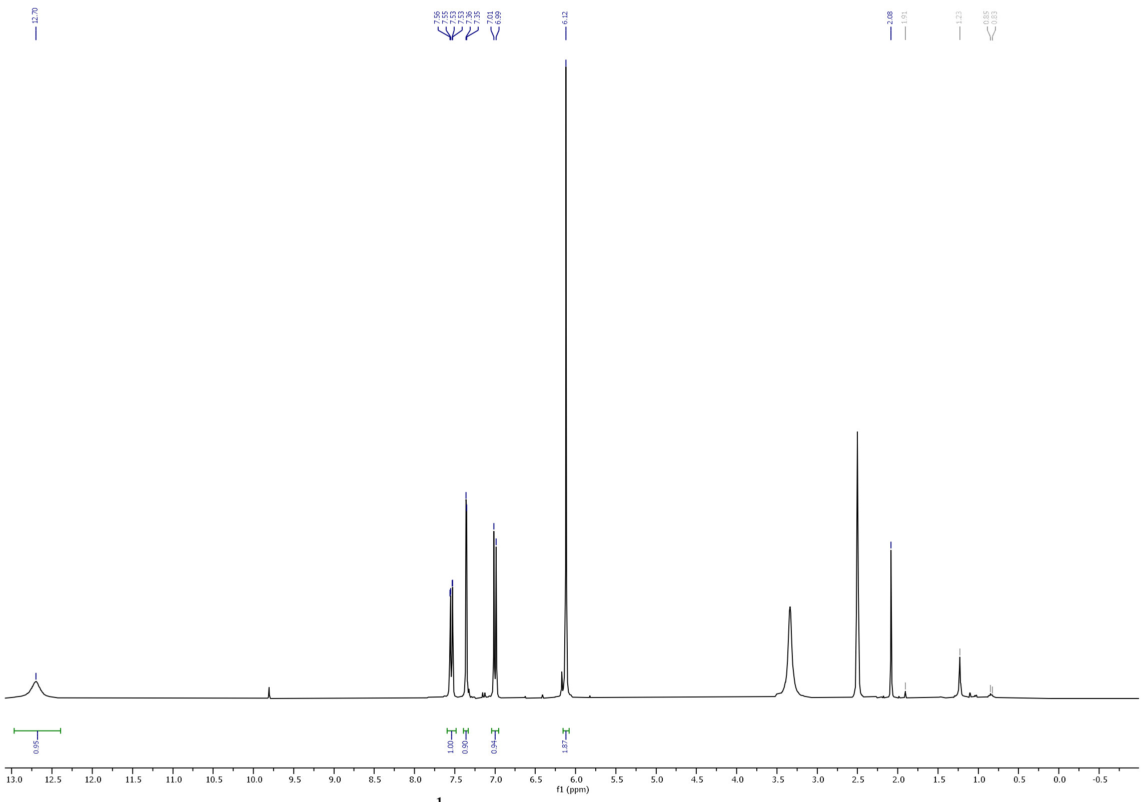

**Fig. S33**. $^{1}$H NMR of compound **12**

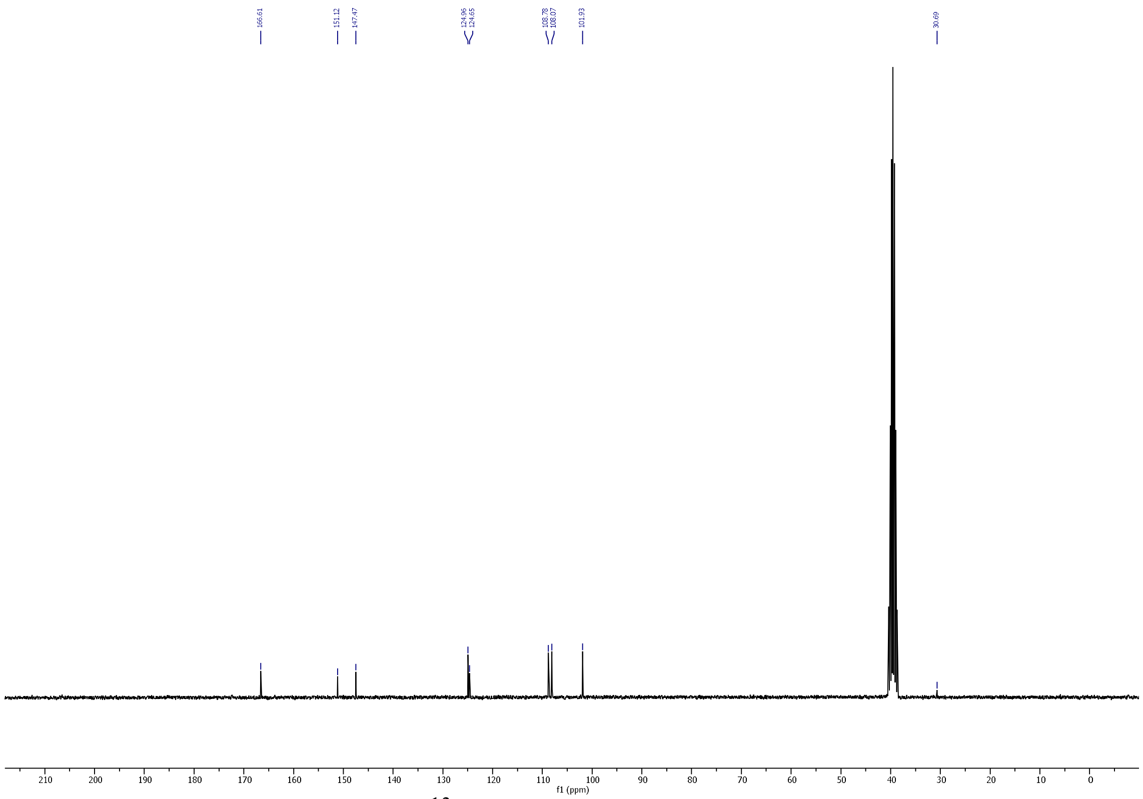

**Fig. S34**. $^{13}$C NMR of compound **12**

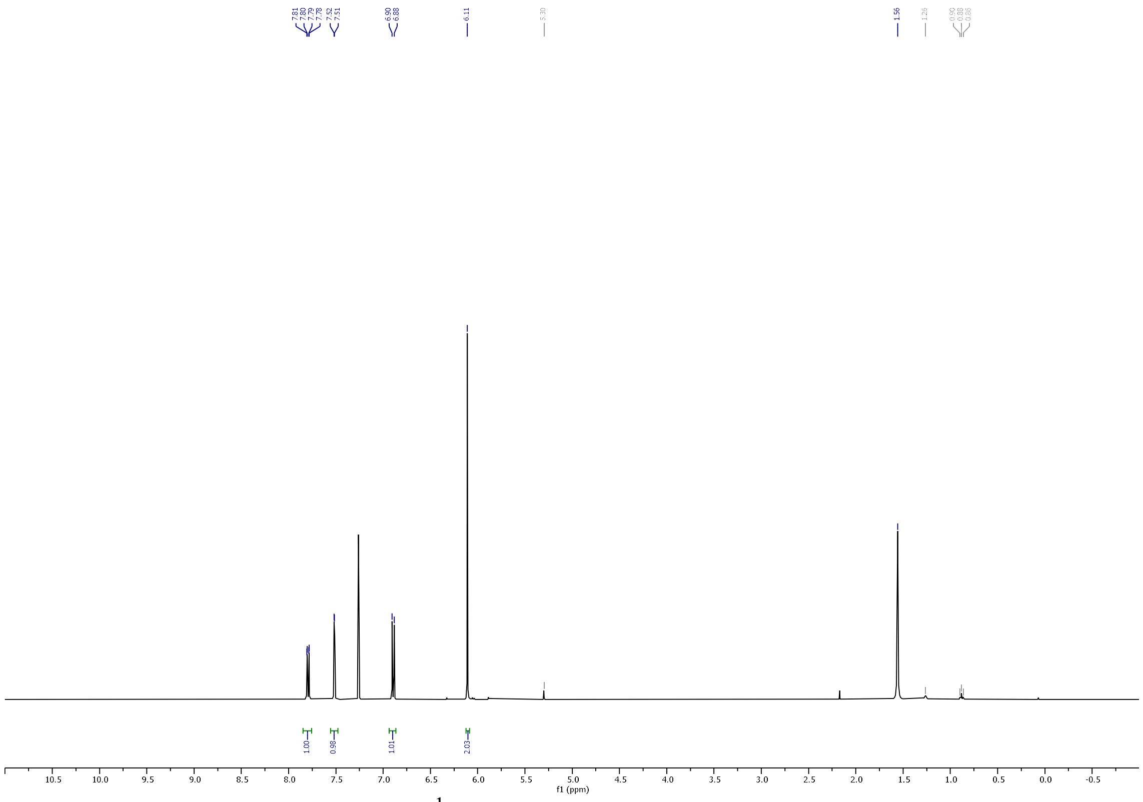

**Fig. S35**. $^{1}$H NMR of compound **13**

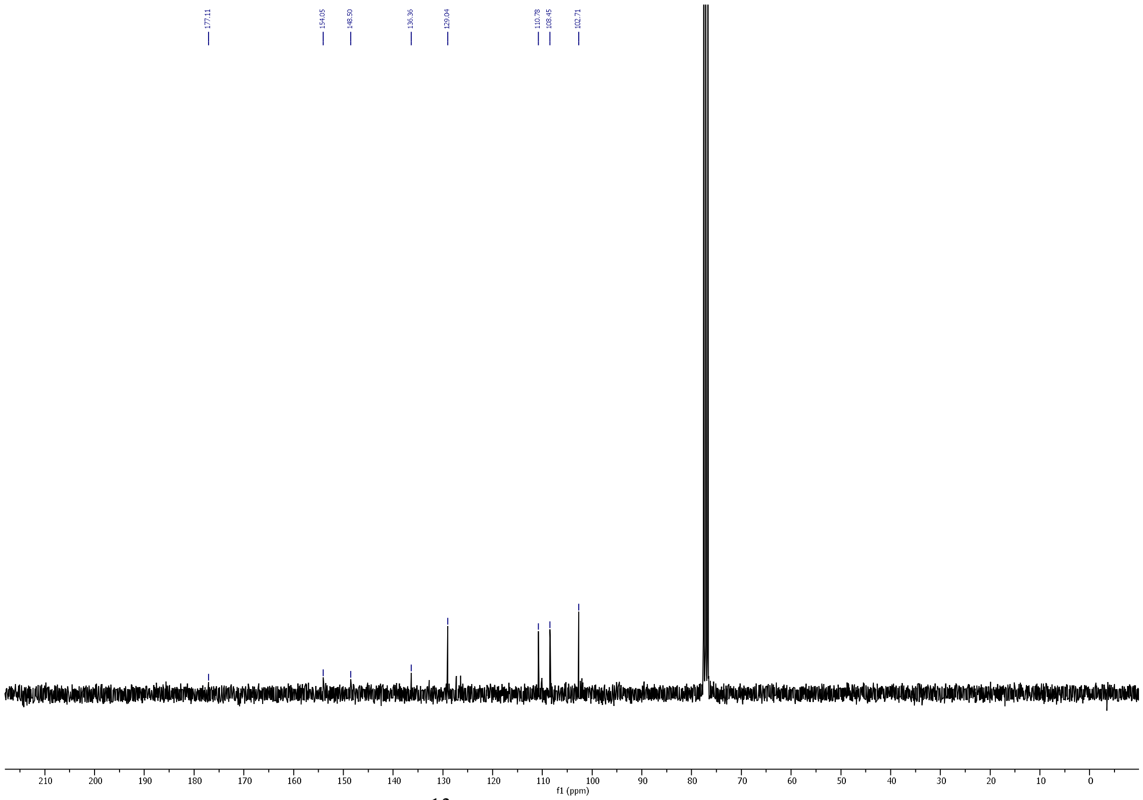

**Fig. S36**. $^{13}$C NMR of compound **13**

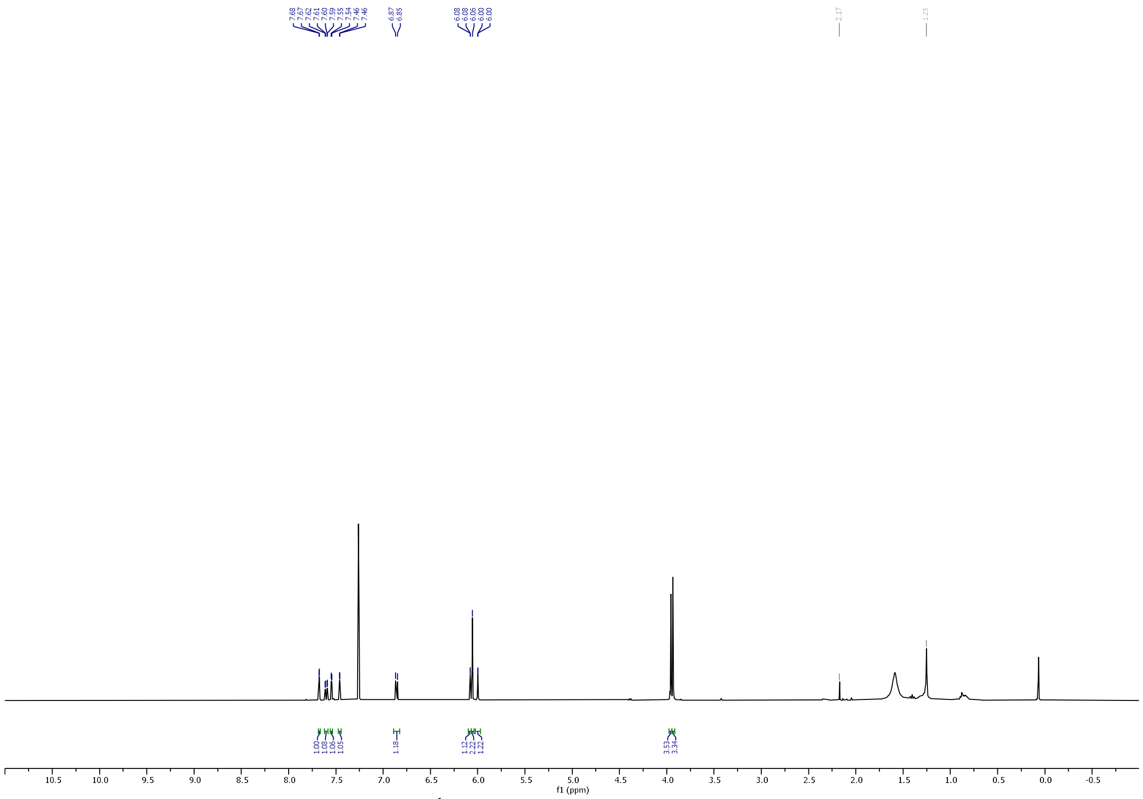

**Fig. S37**. $^{1}$H NMR of compound **14**

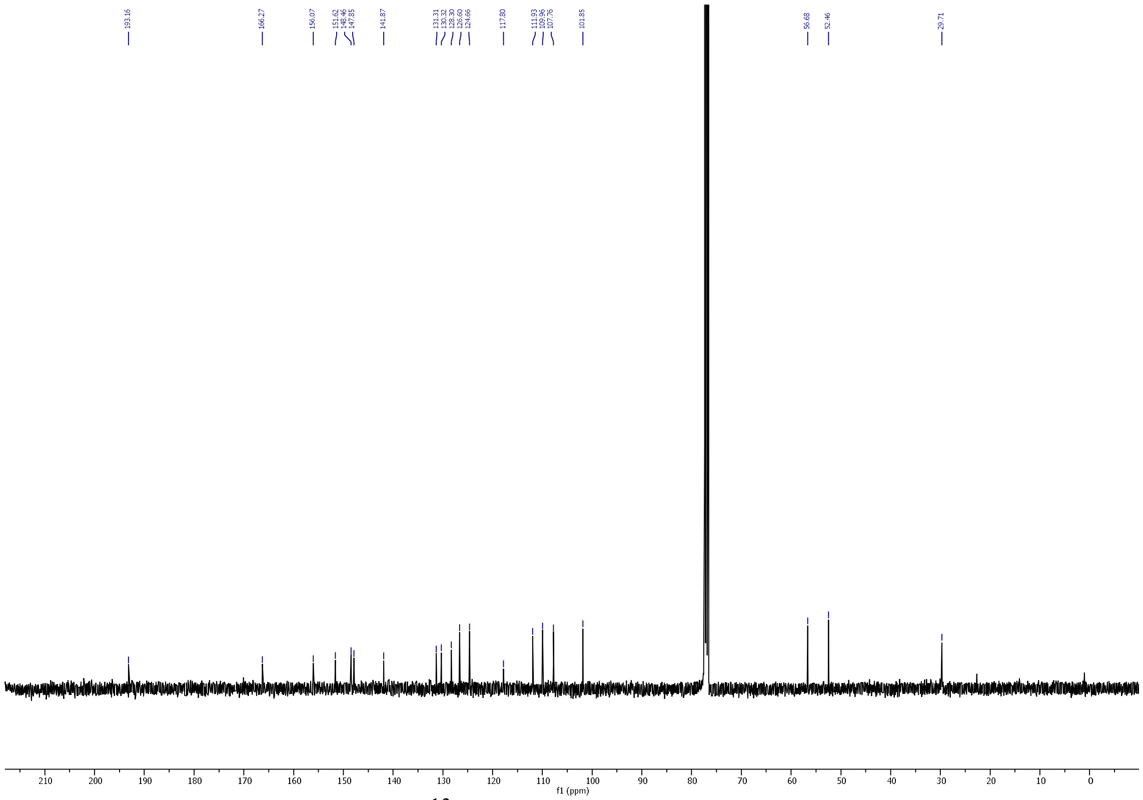

**Fig. S38**. $^{13}$C NMR of compound **14**

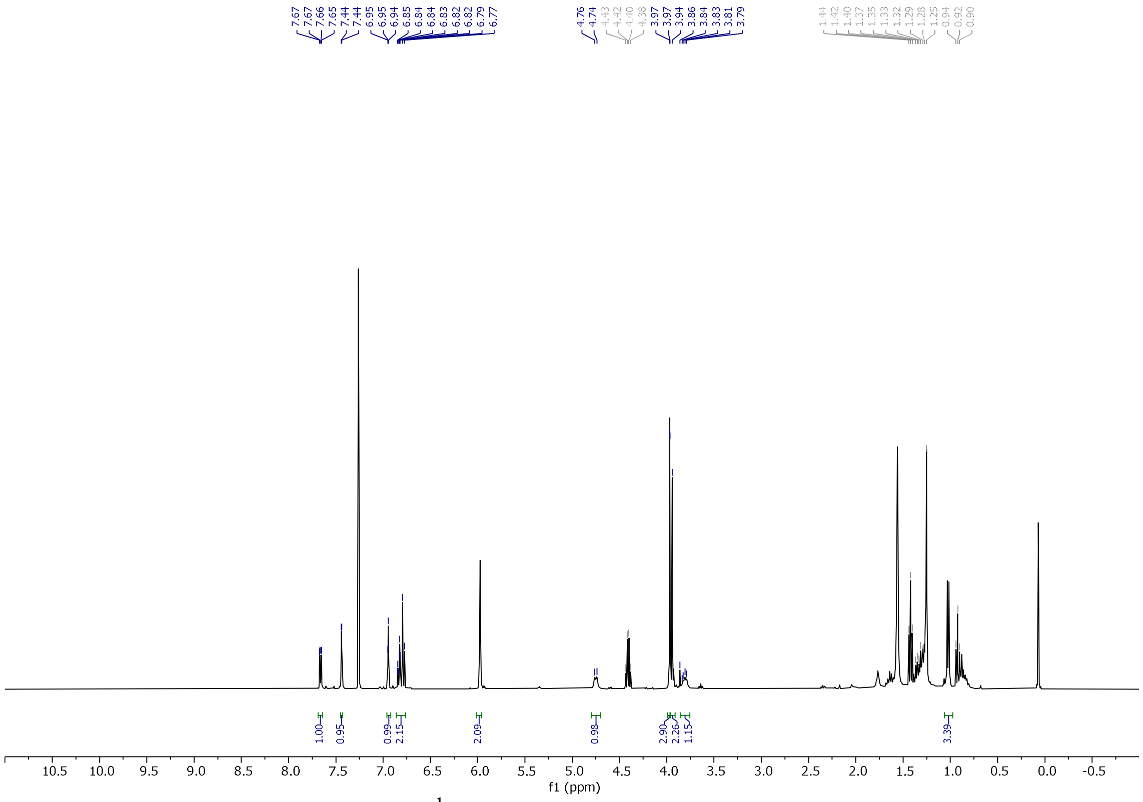

**Fig. S39**. $^{1}$H NMR of compound **15**

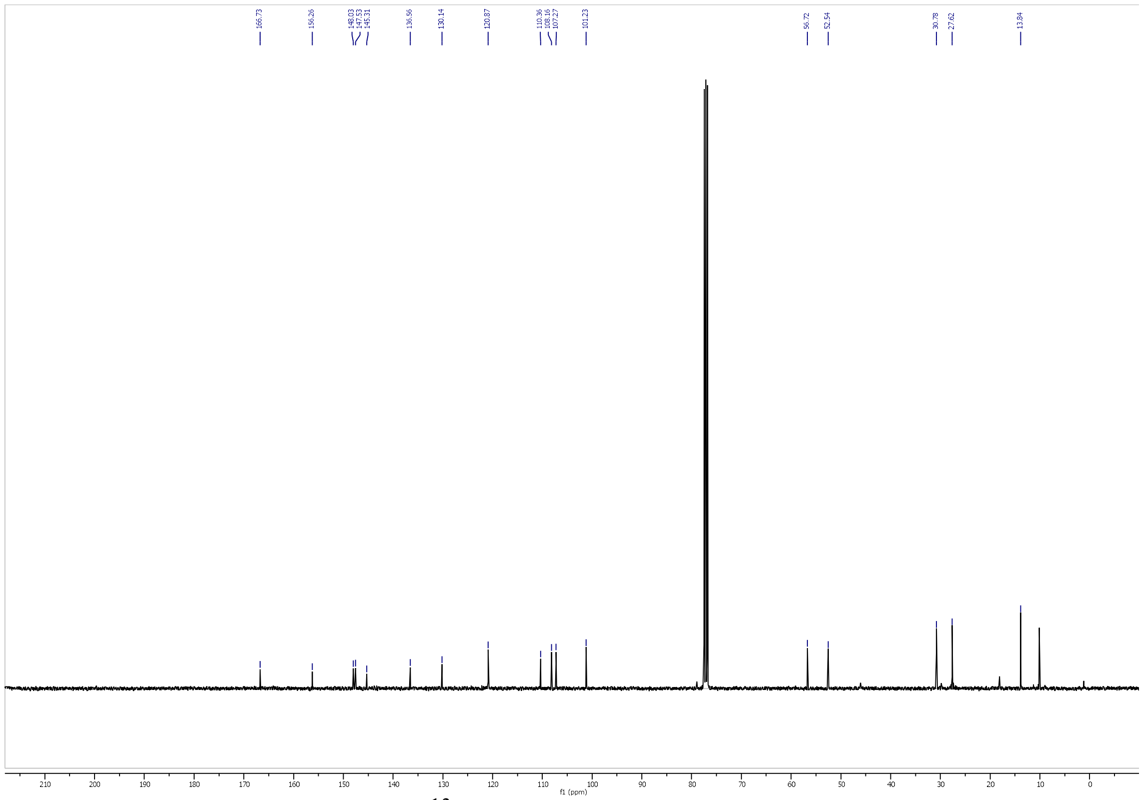

**Fig. S40**. $^{13}$C NMR of compound **15**

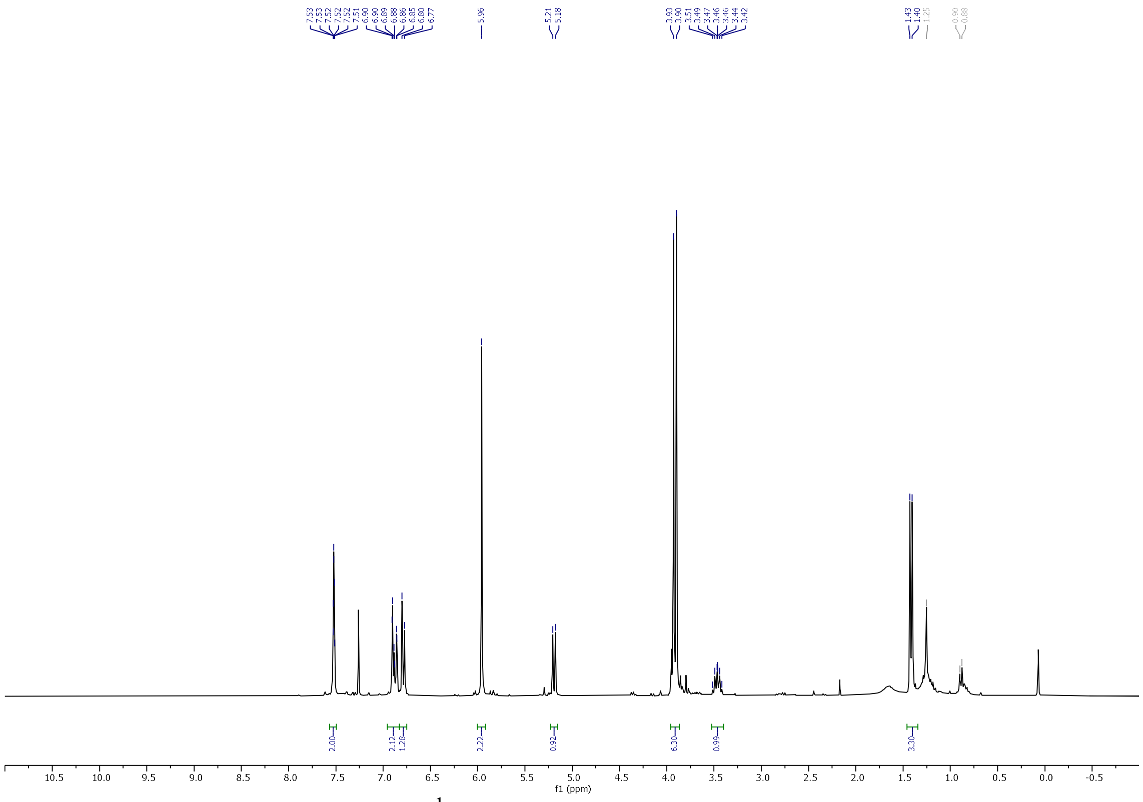

**Fig. S41**. $^{1}$H NMR of compound **16**

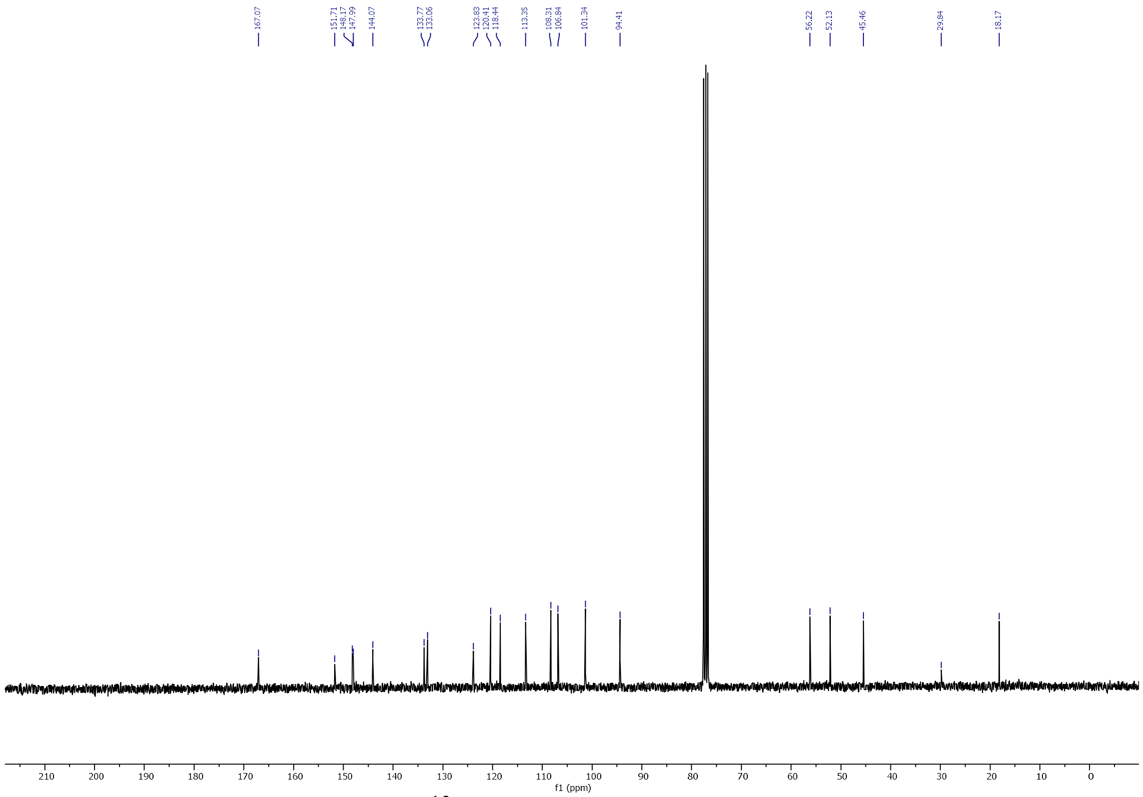

**Fig. S42**. $^{13}$C NMR of compound **16**

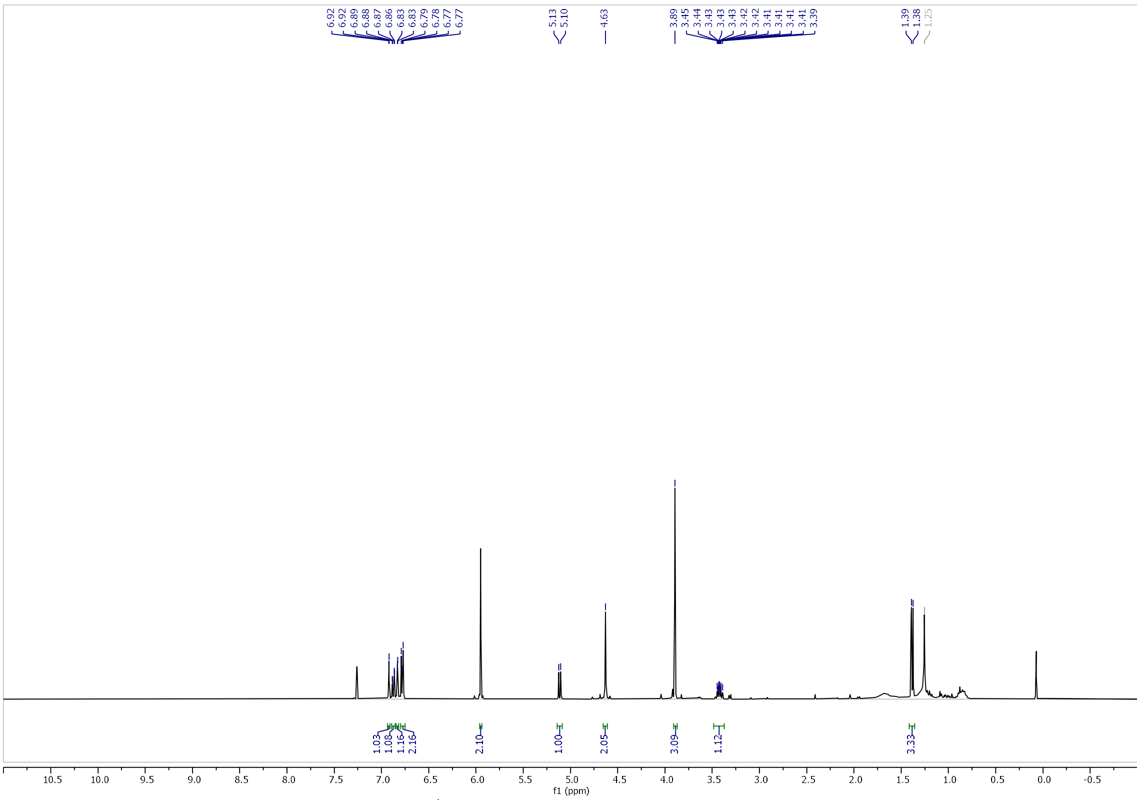

**Fig. S43**. $^{1}$H NMR of compound **17**

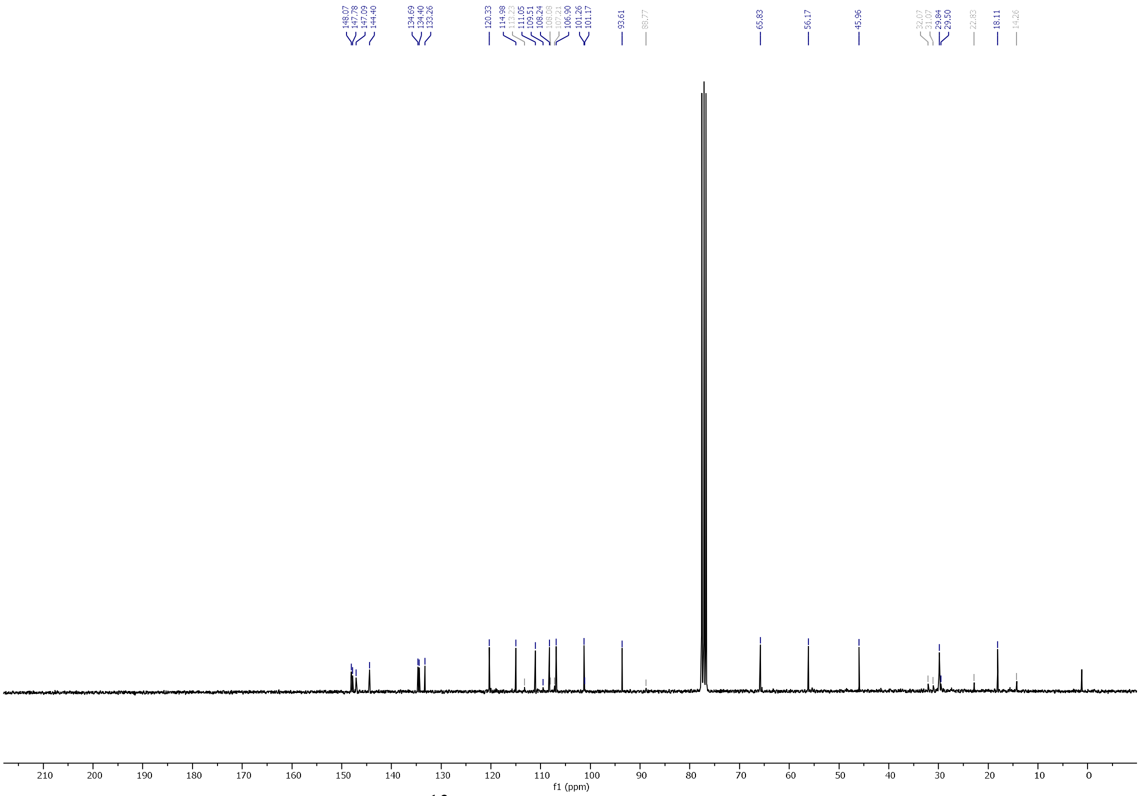

**Fig. S44**. $^{13}$C NMR of compound **17**

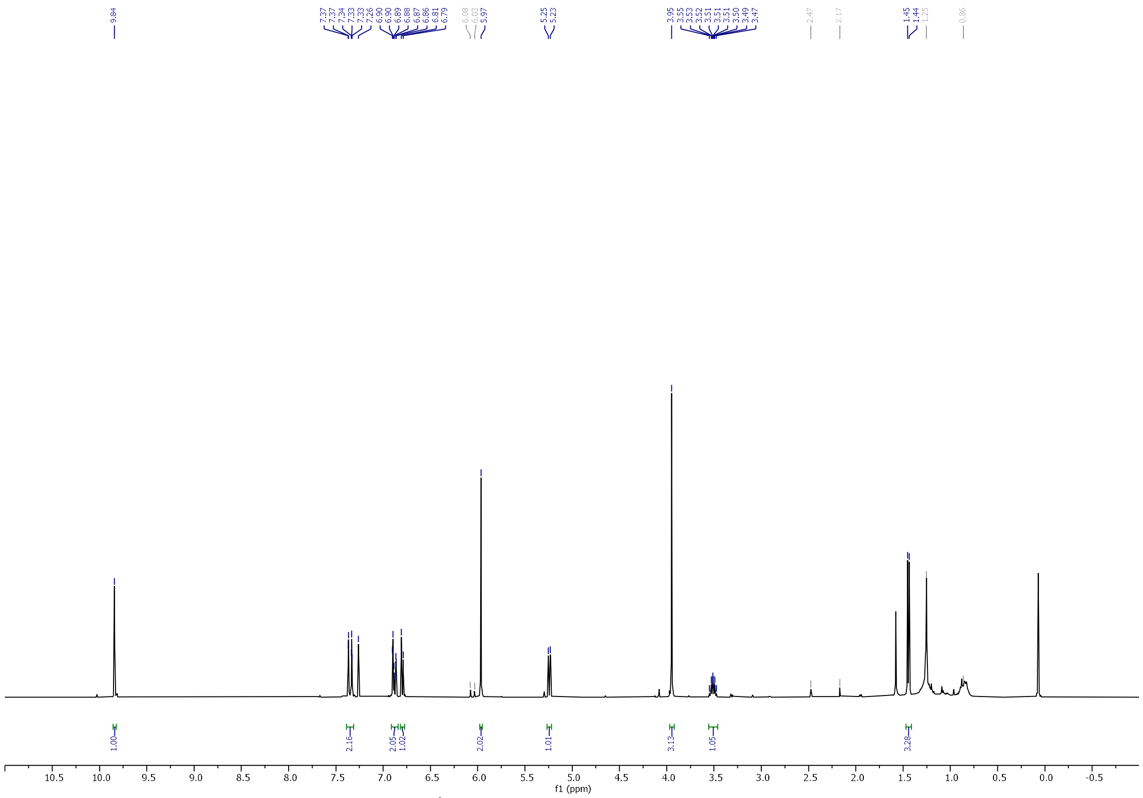


**Fig. S45**. $^{1}$H NMR of compound **18**

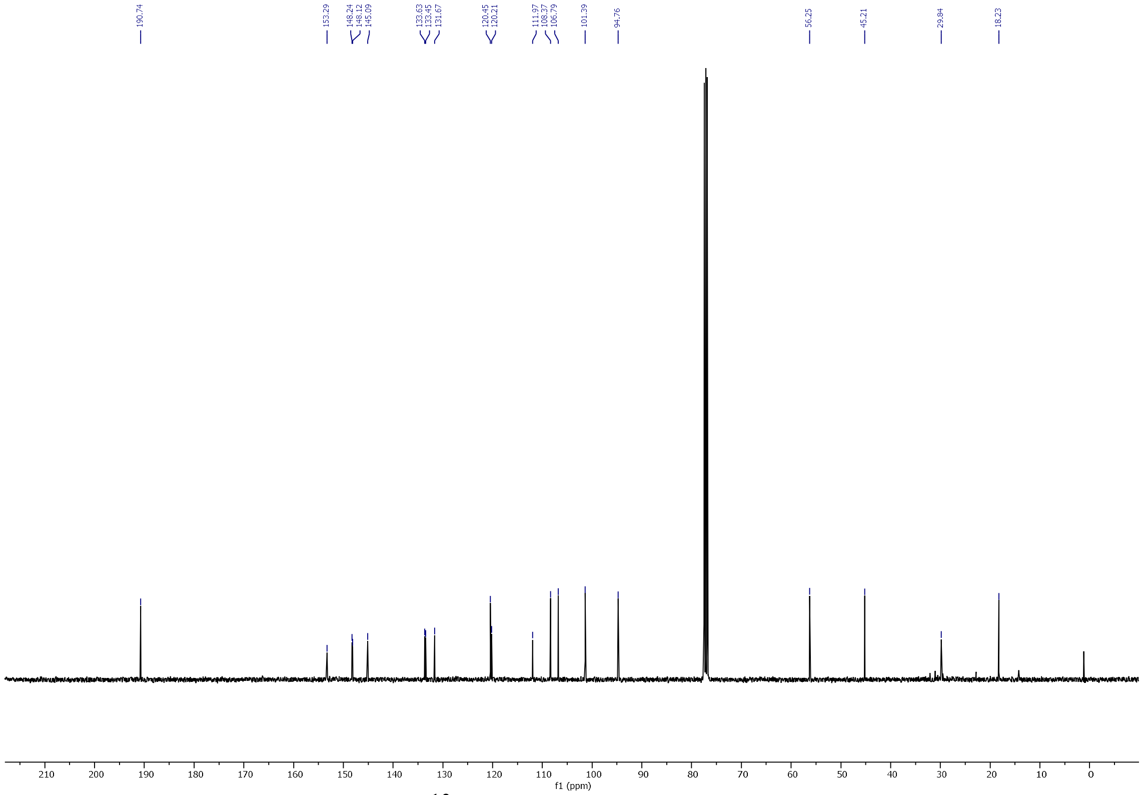


**Fig. S46**. $^{13}$C NMR of compound **18**

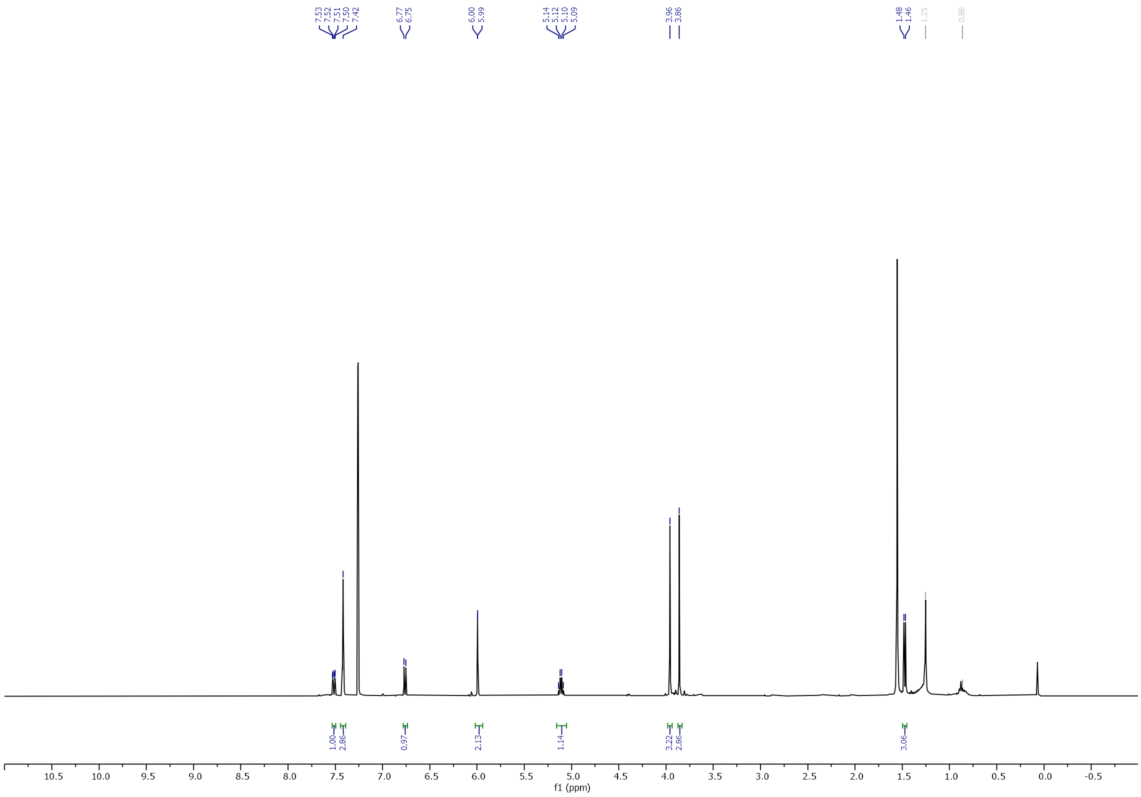


**Fig. S47**. $^{1}$H NMR of ketone **S9**.

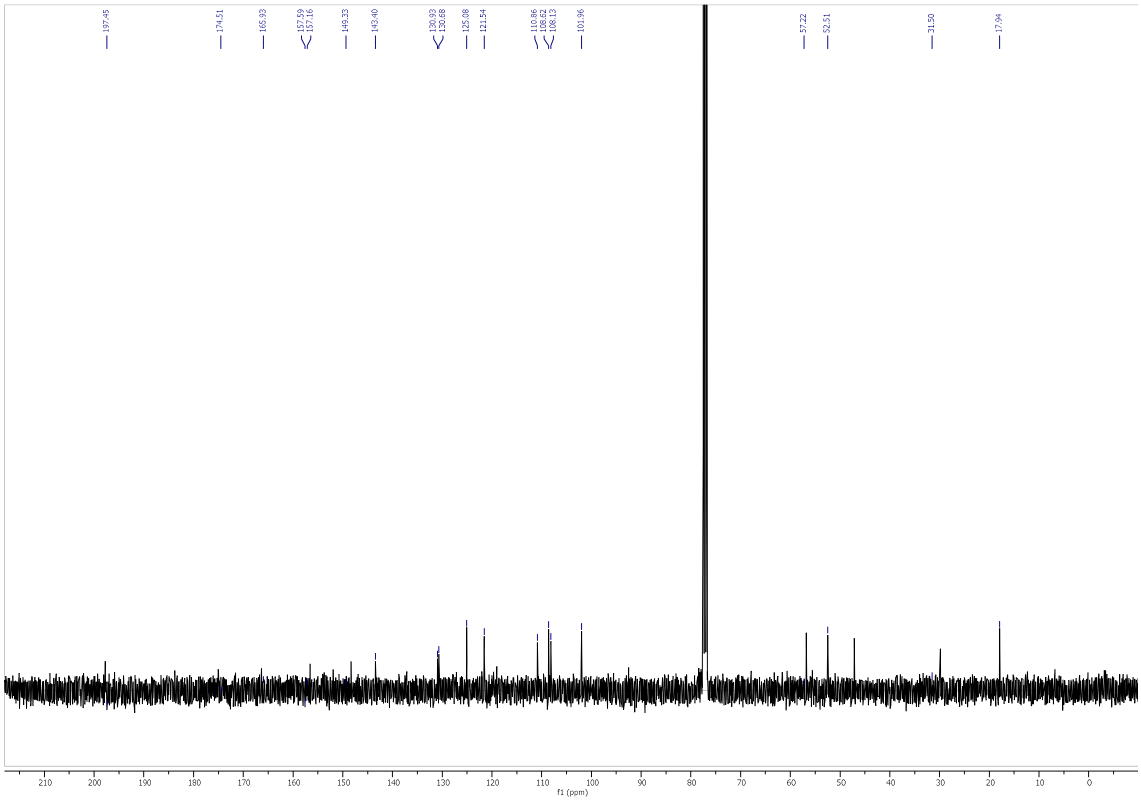


**Fig. S48**. $^{13}$C NMR of ketone from reduction of **S9**.

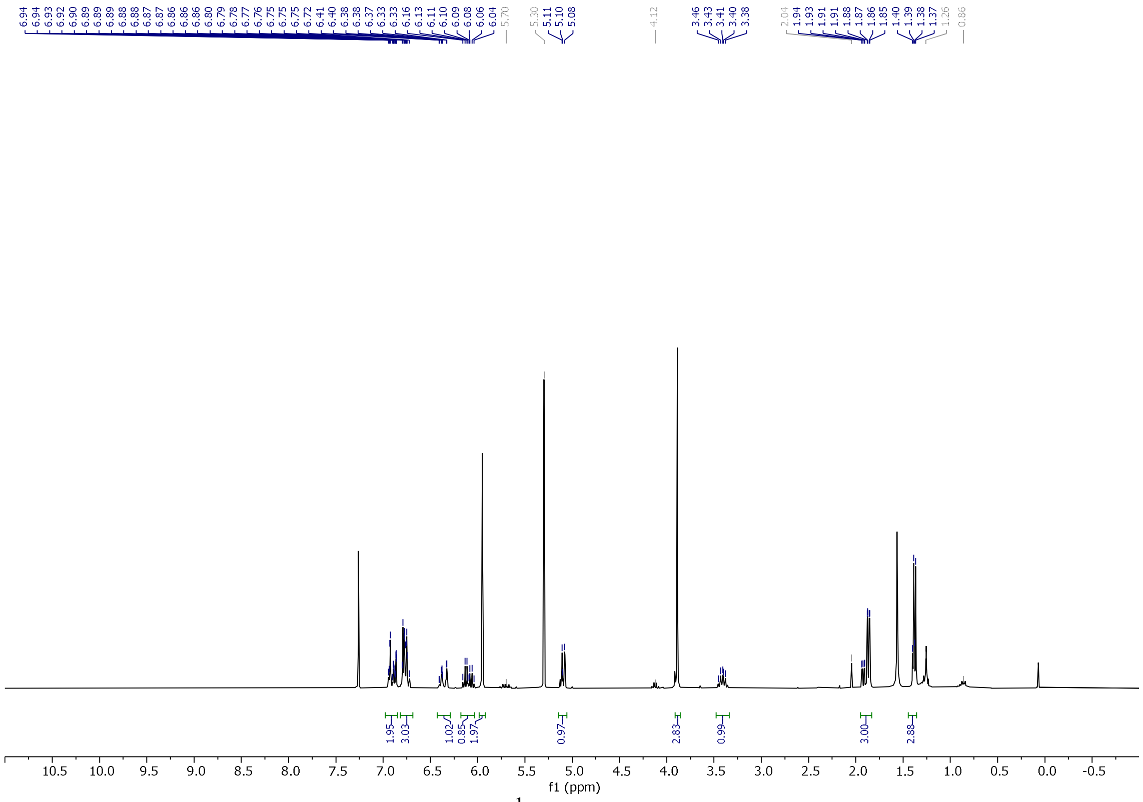

**Fig. S49**. $^{1}$H NMR of **1**

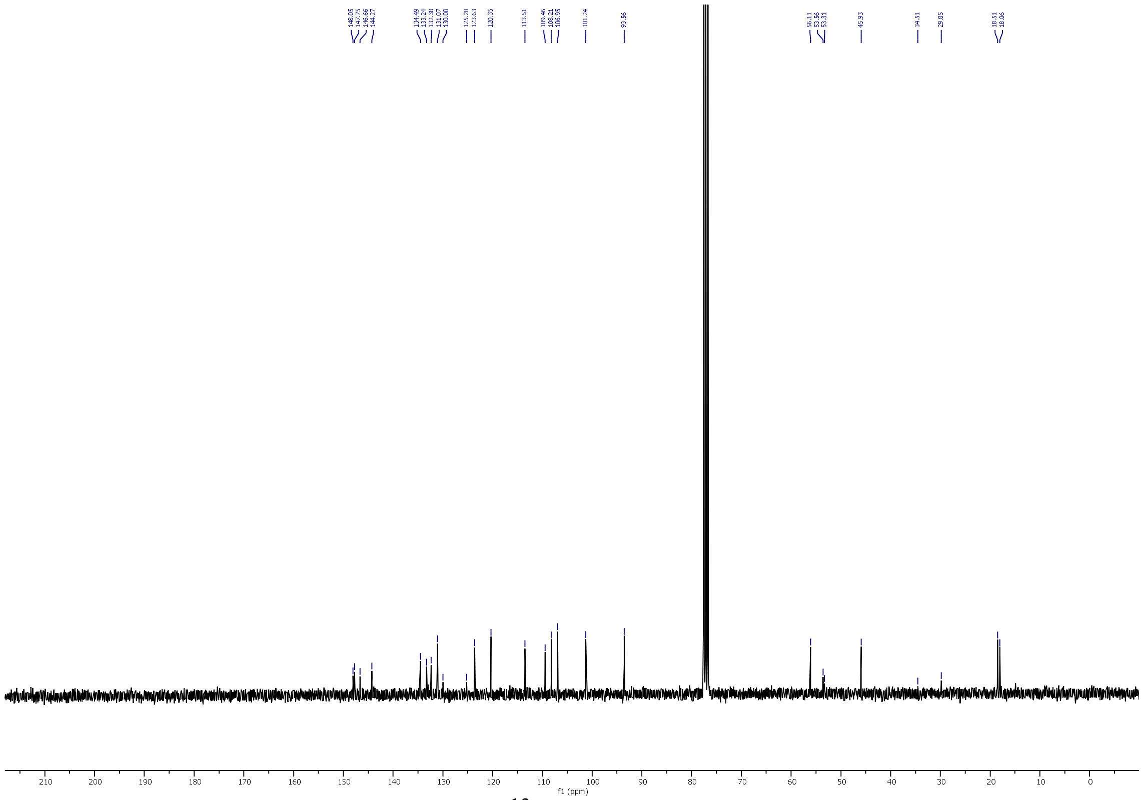

**Fig. S50**. $^{13}$C NMR of **1**

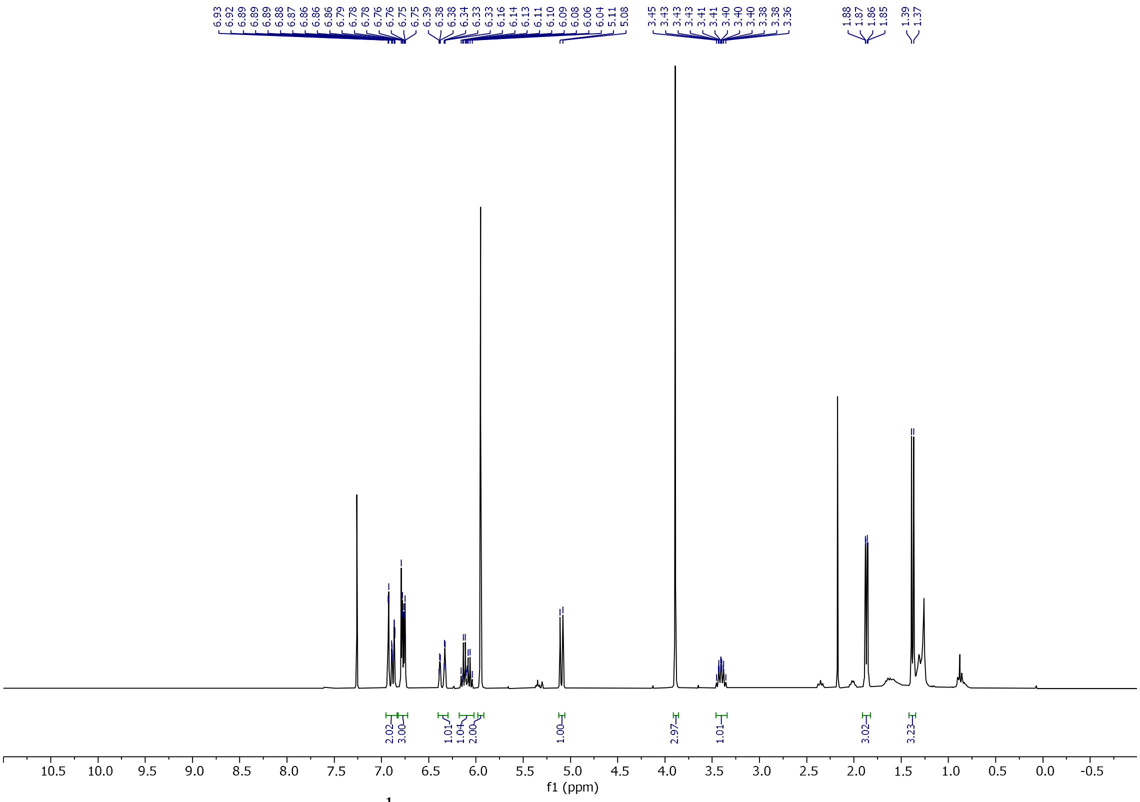


**Fig. S51**. $^{1}$H NMR of commercial Licarin B

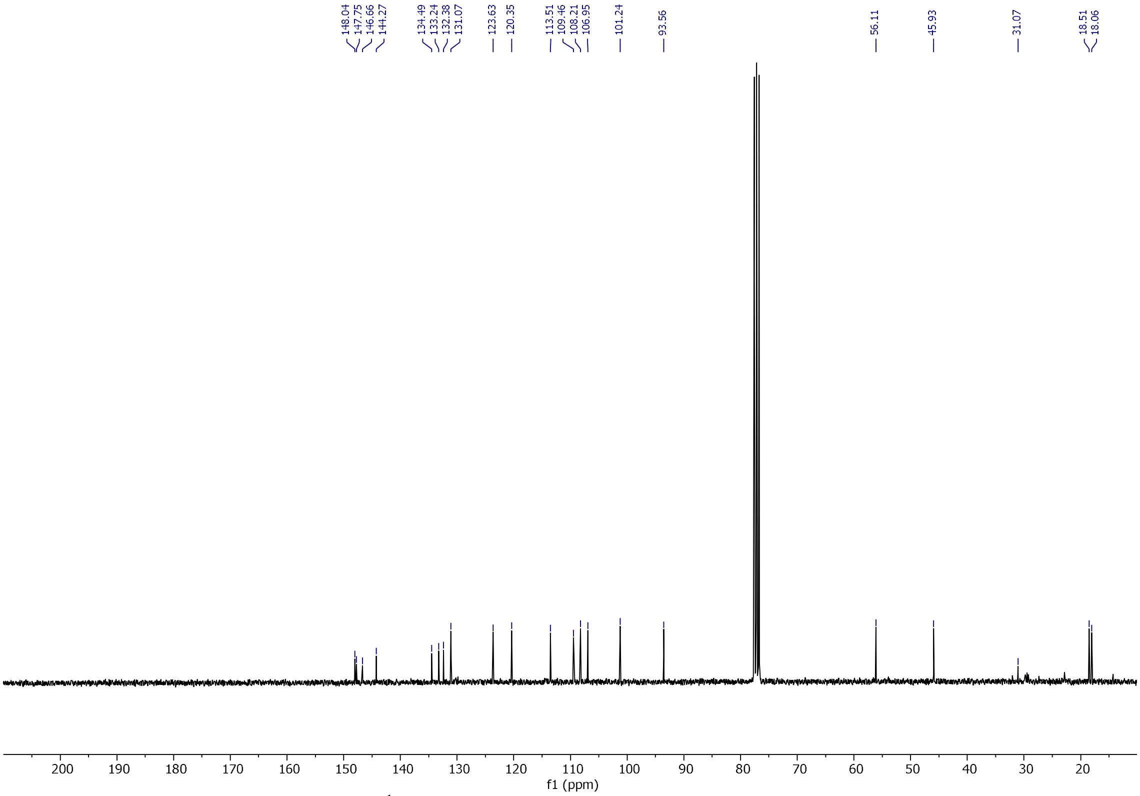


**Fig. S52**. $^{1}$H NMR of commercial Licarin B
